# A lignan from *Alnus japonica* inhibits glioblastoma tumorspheres by suppression of FOXM1

**DOI:** 10.1038/s41598-022-18185-w

**Published:** 2022-08-17

**Authors:** Jin-Kyoung Shim, Seung Hoon Lim, Ji Hye Jeong, Ran Joo Choi, Yoojung Oh, Junseong Park, Sunghee Choi, Junpyo Hong, Seo Jin Kim, Ju Hyung Moon, Eui Hyun Kim, Wan-Yee Teo, Bong Jin Park, Jong Hee Chang, Jae-Ha Ryu, Seok-Gu Kang

**Affiliations:** 1 Department of Neurosurgery, Brain Tumor Center, Severance Hospital, Yonsei University College of Medicine, Seoul, Republic of Korea; 2 Department of Neurosurgery, Kyung Hee University College of Medicine, Seoul, Republic of Korea; 3 Research Institute of Pharmaceutical Sciences and College of Pharmacy, Sookmyung Women’s University, Seoul, Republic of Korea; 4 Precision Medicine Research Center, College of Medicine, The Catholic University of Korea, Seoul, Republic of Korea; 5 Cancer and Stem Cell Biology Program, Duke-NUS Medical School, Singapore, Singapore; 6 Institute of Molecular and Cell Biology, A*STAR, Singapore, Singapore; 7 Department of Medical Science, Yonsei University Graduate School, Seoul, Republic of Korea

**Keywords:** CNS cancer, Translational research, Cancer

## Abstract

Forkhead Box M1 (FOXM1) is known to regulate cell proliferation, apoptosis and tumorigenesis. The lignan, (−)-(2R,3R)-1,4-O-diferuloylsecoisolariciresinol (DFS), from *Alnus japonica* has shown anti-cancer effects against colon cancer cells by suppressing FOXM1. The present study hypothesized that DFS can have anti-cancer effects against glioblastoma (GBM) tumorspheres (TSs). Immunoprecipitation and luciferase reporter assays were performed to evaluate the ability of DFS to suppress nuclear translocation of β-catenin through β-catenin/FOXM1 binding. DFS-pretreated GBM TSs were evaluated to assess the ability of DFS to inhibit GBM TSs and their transcriptional profiles. The in vivo efficacy was examined in orthotopic xenograft models of GBM. Expression of FOXM1 was higher in GBM than in normal tissues. DFS-induced FOXM1 protein degradation blocked β-catenin translocation into the nucleus and consequently suppressed downstream target genes of FOXM1 pathways. DFS inhibited cell viability and ATP levels, while increasing apoptosis, and it reduced tumorsphere formation and the invasiveness of GBM TSs. And DFS reduced the activities of transcription factors related to tumorigenesis, stemness, and invasiveness. DFS significantly inhibited tumor growth and prolonged the survival rate of mice in orthotopic xenograft models of GBM. It suggests that DFS inhibits the proliferation of GBM TSs by suppressing FOXM1. DFS may be a potential therapeutic agent to treat GBM.

## Introduction

Glioblastoma (GBM), the most common primary malignant tumor in the brain, is highly aggressive^1^. Despite intensive treatment with surgery, chemotherapy and radiotherapy, patients with GBM have a high mortality rate and poor prognosis^1–3^. To date, furthermore, relatively little is known about the cells from which GBM originates or the genetic mutations that accompany GBM tumorigenesis, so GBM still remains a difficult disease to conquer^4,5^. However, previous studies reported that GBM tumorsphere (TS), characterized by GBM resident cells in culture has a treatment refractory properties, as well as a stemness profile and an ability to form tumors, and it has been shown to have value for clinical use^6,7^.

Forkhead Box M1 (FOXM1) is a member of the Forkhead box transcription factor family that has been shown to regulate DNA damage repair, cellular proliferation, apoptosis, angiogenesis and tumorigenesis^8^. Expression of FOXM1 is significantly elevated in many human tumors, including non-small cell lung cancer, breast cancer, basal cell carcinoma, hepatocellular carcinoma, pancreatic cancer, prostate cancer, colon cancer, medulloblastoma and GBM^9–13^. High expression of FOXM1 in glioma cells enhances tumorigenicity, invasiveness and angiogenesis in GBM animal models^14–16^. Furthermore, FOXM1 was found to regulate the Wnt/β-catenin signaling pathway by promoting the nuclear translocation of β-catenin ^17,18^.

*Alnus japonica* (Betulaceae) is a plant used as a medicinal herb in east Asia. This plant contains pharmacologically active compounds, including the isolated lignan, (−)-(2R,3R)-1,4-*O*-diferuloylsecoisolariciresinol (DFS), which was found to reduce the viability of colon cancer cells and to interrupt the cell cycle. We previously reported that DFS interfere with β-catenin translocation into the nucleus, and to subsequently reduce the levels of expression of β-catenin mediated genes through suppression of FOXM1 protein expression in colon cancer^19^. DFS also induced autophagy and endoplasmic reticulum (ER) stress in prostate and colon cancer cells^20^. FOXM1/β-catenin interactions can also regulate the stemness and tumorigenicity of glioma stem cells^18^. Because GBM TSs are resistant to conventional treatments such as radiotherapy and chemotherapy, they have been considered a good platform for testing the therapeutic efficacy of drug candidates. We have tried to find promising anti-GBM agent from medicinal plants such as *A. japonica*. Relatively little is known, however, about the effects of the DFS in GBM TSs.

The present study hypothesized that DFS has the ability to block β-catenin translocation into the nucleus by suppressing the formation of FOXM1/β-catenin complex in GBM TSs. In addition, the anti-cancer potential of DFS against GBM was evaluated using GBM TSs and an orthotopic xenograft mouse model.

## Results

### Expression level of FOXM1 and β-catenin mRNAs in GBM tissues

GBM tissue specimens were obtained from 49 patients who underwent surgery for GBM from May 2009 to December 2016, and among 49 patients, 8 normal brain tissue specimens were also obtained. All the specimens were from the patients diagnosed with GBM for the first time, and tissues from the patients with recurred GBM were excluded.

**Figure 1:**
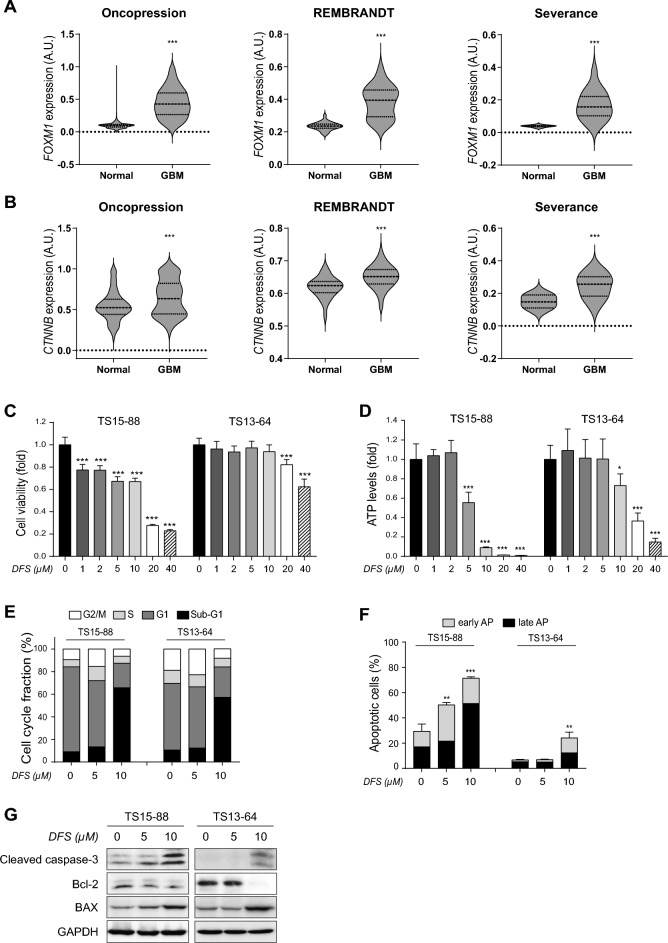
Expression of FOXM1 and β-catenin mRNAs and proteins in GBM tissues and inhibitory effects of DFS on cell survival. (**A**,**B**) Levels of expression of (**A**) FOXM1 and (**B**) β-catenin (CTNNB1) mRNAs and comparison with public datasets. 865 GBM tissues and 723 normal brain tissues in the dataset of Oncopression, while 228 GBM tissues and 28 normal brain tissues in the dataset of REMBRANDT. (**C**) Cell viability and (**D**) ATP levels measured after 72 h of treatment with the indicated concentrations of DFS. Results are expressed as the means ± SD (n = 5). (**E**,**F**) GBM TSs were treated with DFS for 72 h, and (**E**) cell cycle fractions and (**F**) apoptotic cell populations analyzed by FACS. (**G**) Expression of apoptosis-associated proteins measured by western blotting after treatment with DFS for 72 h. Differences among groups were compared by one-way ANOVA with Tukey’s post hoc test for multiple comparisons; *P < 0.05, **P < 0.01, ***P < 0.001.

Similar to previous reports^21,22^, the levels of expression of FOXM1 and β-catenin (CTNNB1) mRNAs were significantly higher in 49 of GBM tissues than in 8 of normal tissues, and public datasets, Oncopression and REMBRANDT, showed similar results of FOXM1 and β-catenin (CTNNB1) expression level (Fig. 1A,B).

### DFS inhibits GBM TSs viability and ATP levels in vitro

To assess the mechanism of action of DFS against GBM TSs, we evaluated the effects of DFS on cell viability and ATP levels. WST assays confirmed that DFS dose-dependently reduced cell viability in GBM TSs (Fig. 1C), as well as reducing ATP levels in a concentration dependent manner (Fig. 1D). Based on these results, DFS concentrations of 5 µM and 10 µM were selected for further studies. DFS treatment significantly increased the percentage of GBM TSs in sub-G1 phases (more than 55%) compared with control (about 10%) (Fig. 1E), as well as markedly increasing the percentage of cells in early stages of apoptosis (Fig. 1F). DFS also altered the levels of apoptotic markers, such as Bcl-2, BAX and cleaved caspase-3, indicating that DFS induced cell apoptosis (Fig. 1G). Taken together, these results indicated that DFS decreased cell proliferation while inducing cell death of GBM TSs at dose of higher than 5 µM.

### DFS treatment suppresses stemness and invasiveness of GBM TSs

**Figure 2:**
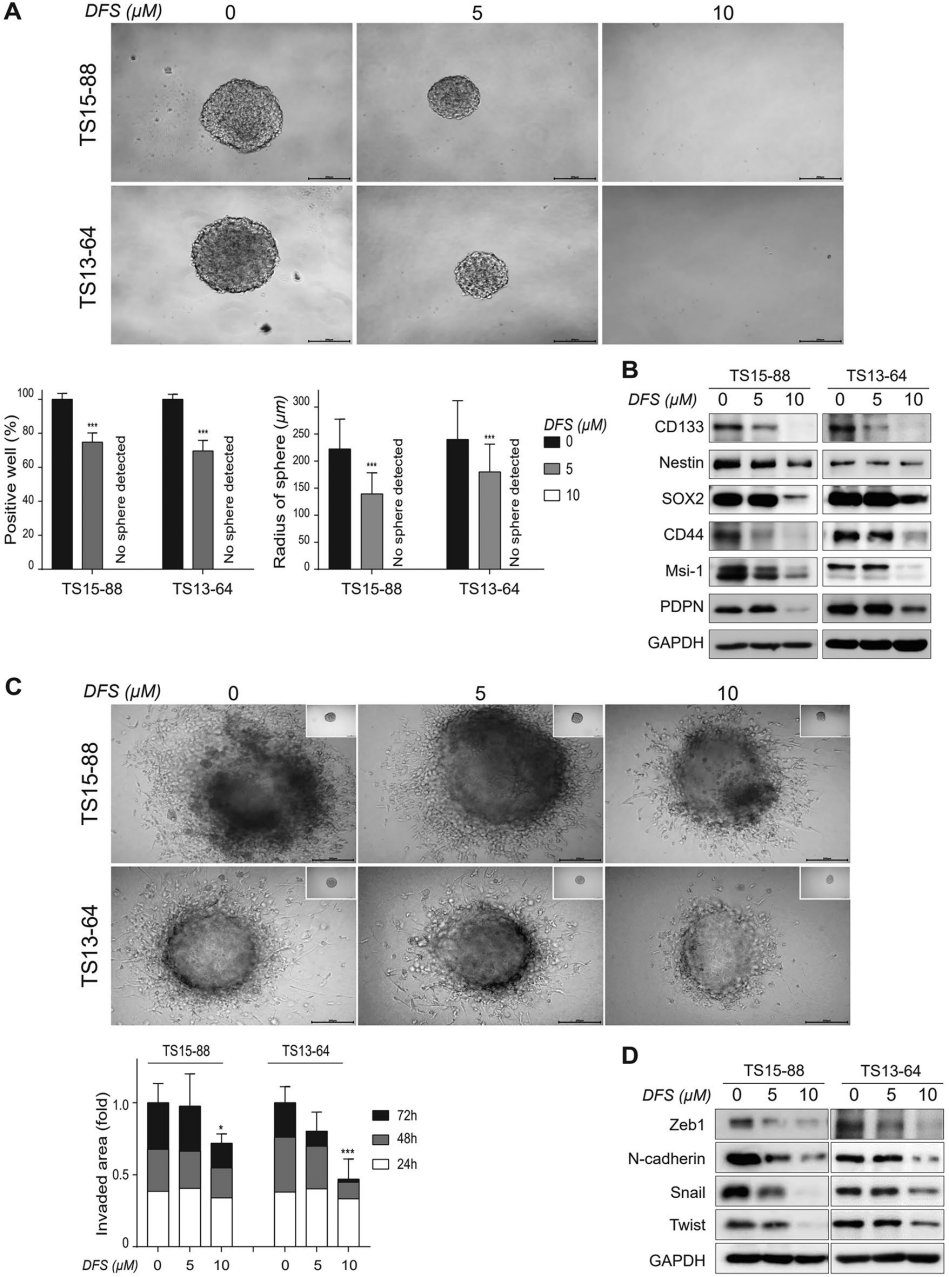
Inhibitory effects of DFS on stemness and invasiveness of GBM TSs. (**A**) Stemness was determined by Sphere formation assay. Cells were treated with DFS for 3 weeks and the percentages of sphere-positive wells and sphere radii were calculated. (**B**) GBM TSs were treated with DFS for 72 h and stemness-associated protein levels were determined by western blotting. (**C**) GBM TSs were cultured on matrigel/collagen matrix with DFS for 72 h, and invasiveness was quantified by measuring areas of migration. (**D**) Western blotting assays of the expression of EMT-related proteins. All Images are × 10 original magnification (scale bar = 200 μm). Differences among groups were compared by one-way ANOVA with Tukey’s post hoc test; means ± SD; *P < 0.05, **P < 0.01, ***P < 0.001, between indicated groups or compared with controls.

Stemness and invasiveness are critical aspects of neural stem cells. We therefore measured the effects of DFS on stemness and invasiveness of GBM TSs. Sphere formation assays evaluate the stemness of GBM TSs, a characteristic essential for the self-renewal, proliferation and multipotency of cells. Treatment of GBM TSs with 10 µM DFS for 3 weeks significantly reduced the percentage of sphere-positive wells and sphere radii (Fig. 2A), as well as reducing the levels of expression of stemness-related marker proteins, including CD133, Nestin, Sox2, CD44, Msi-1 and PDPN (Fig. 2B). Treatment with 10 µM DFS also significantly inhibited the invasive capacity of GBM TSs, as determined by 3D matrigel invasion assays (Fig. 2C), as well as markedly reducing the expression of invasiveness-associated marker proteins, including Zeb1, N-cadherin, Snail and Twist (Fig. 2D). Taken together, these results suggest that DFS significantly suppressed the stemness and invasiveness of patient-derived GBM TSs.

### DFS reduces β-catenin nuclear translocation by suppressing FOXM1 expression

Prior to the investigation of inhibitory effect of DFS on nuclear translocation of β-catenin, the marker proteins in the cytosol and nucleus were identified using PARP and β-tubulin to confirm nucleocytoplasmic separation. As a result, PARP positive/β-tubulin negative in the nucleus; PARP negative/β-tubulin positive in the cytosol, were identified and therefore validated the nucleus/cytosol fraction.

**Figure 3:**
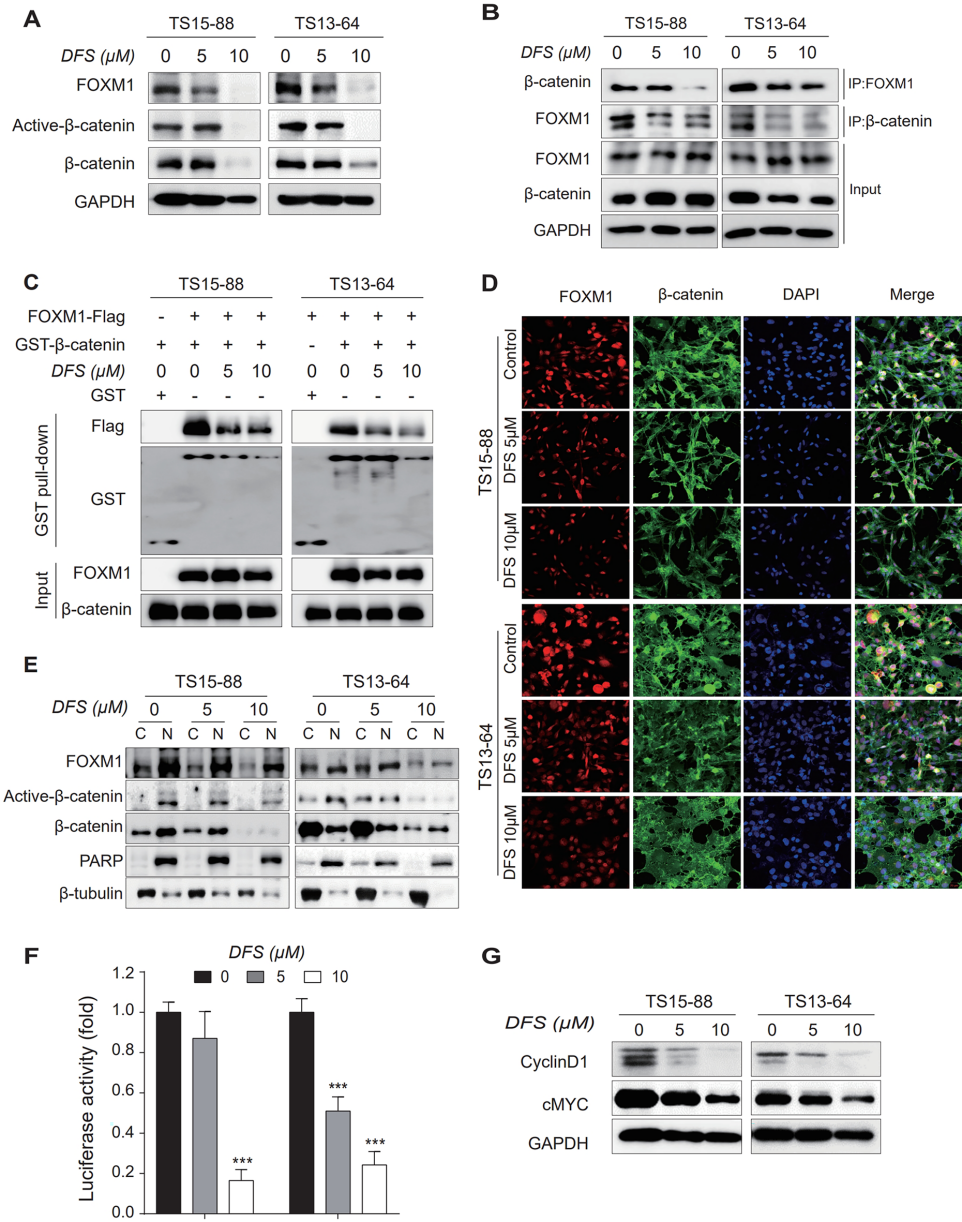
Inhibition of FOXM1/ β-catenin interaction by DFS. (**A**) GBM TSs were treated with DFS for 72 h and the protein levels of FOXM1, active-β-catenin and total β-catenin were determined by western blotting. (**B**) DFS-treated GBM TSs were immunoprecipitated with anti-FOXM1 and β-catenin antibody, and endogenous expression of FOXM1 and β-catenin proteins were determined by western blotting to determine interactions between them. (**C**) GST pull-down assay to confirm whether FOXM1 and β-catenin interactions are direct or indirect. (**D**) Immunofluorescence to determine whether DFS affects the nuclear translocation of β-catenin (magnification × 20). (**E**) Levels of FOXM1 and β-catenin proteins in the cytosolic and nuclear fractions of GBM TSs determined by western blotting. The cytosolic and nuclear fractions are denoted by C and N, respectively. Cytosolic marker: β-tubulin; nuclear marker: PARP. (**F**) Measurements of β-catenin/TCF signaling activity in GBM TSs transfected with TOPflash or FOPflash luciferase vector. (**G**) Expression of proteins encoded by β-catenin downstream genes (cyclin D1, cMYC) measured by western blotting. Differences among groups were compared by one-way ANOVA with Tukey’s post hoc test; means ± SD; *P < 0.05, **P < 0.01, ***P < 0.001, between indicated groups or compared with controls.

To test the inhibitory effects of DFS on FOXM1/β-catenin interactions, proteins encoded by β-catenin pathway-related genes were quantified by western blotting. DFS significantly reduced the levels of FOXM1, active-β-catenin and total β-catenin expression in whole-cell lysate of GBM TSs (Fig. 3A) at 10 µM. IP analyses showed that DFS suppressed the interaction between FOXM1 and β-catenin in GBM TSs (Fig. 3B). We further clarified whether β-catenin and FOXM1 interaction is a direct interaction by performing a series of GST pull-down assays using purified, bacterially produced GST-β-catenin and Flag-tagged FOXM1 lysate from TS15-88 and TS13-64. Our results show that the decreased binding of β-catenin and FOXM1 in a dose-dependent manner after treatment of DFS at a concentration of 5 and 10 μM (Fig. 3C). Surface plasmon resonance (SPR) analysis was performed to confirm binding affinity of β-catenin and FOXM1 by DFS. Our results indicated that the recombinant human β-catenin protein bound FOXM1 in a dose-dependent manner by treatment of DFS concentrations from 12.5 to 50 μM (Supplementary 1). Immunofluorescence (IF) and subcellular fraction revealed that the overall expression of FOXM1 and β-catenin was reduced, and most of β-catenin was expressed in the cytosol and less in the nucleus by DFS (Fig. 3D,E). We also confirmed quantitatively that DFS decreased the fluorescence intensity of FOXM1 and b-catenin (Supplementary 2a), and that the nuclear expression of b-catenin was decreased rather than the cytosolic expression compared to the control group (Supplementary 2b). In addition, DFS markedly reduced TCF/LEF signaling activity in GBM TSs in a dose-dependent manner (Fig. 3F), as well as reducing the expression of cyclin D1 and cMyc, which are regulated by FOXM1/β-catenin forming a complex with the TCF/LEF transcription factor (Fig. 3G). Taken together, these results indicate that DFS inhibited the binding of FOXM1 and β-catenin, thereby inhibiting the formation of complexes with TCF/LEF and sub-steps. In control and DFS-treated GBM TSs, we used cycloheximide to inhibit protein synthesis and looked at FOXM1 protein stability. Our findings revealed that when CHX and DFS were combined, the protein level of FOXM1 decreased much more than when CHX alone was used, indicating that DFS reduced the lifespan of FOXM1 protein and lowered protein stability (Supplementary 3). Thus, DFS decreases the stability of FOXM1 protein by inducing protein degradation. A schematic figure summarizing the whole process of this experiment is described in Supplementary 4.

### Transcriptional profiles following DFS treatment

**Figure 4:**
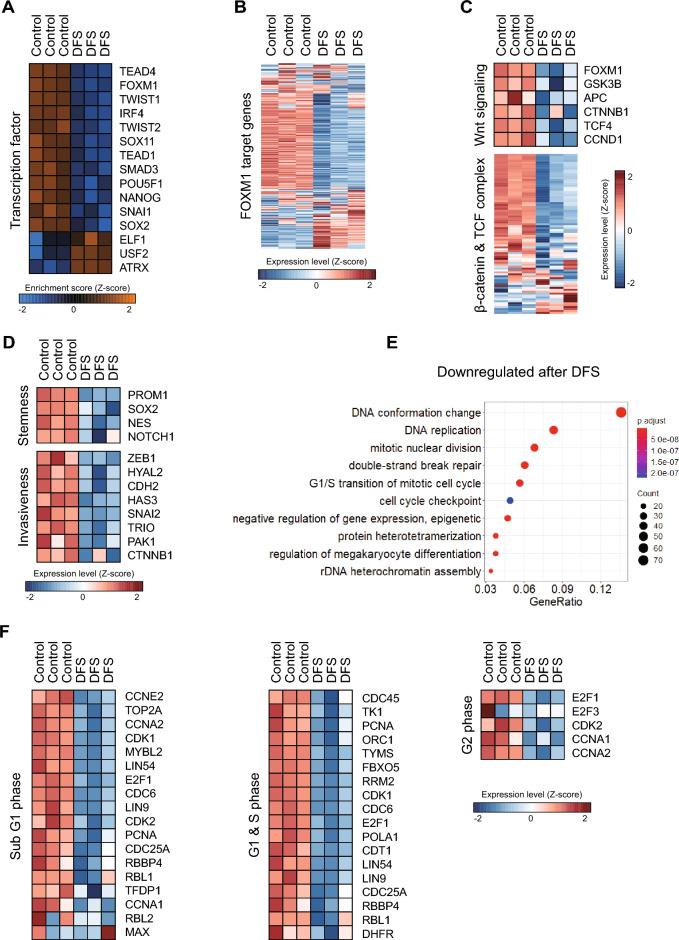
Effects of DFS on transcriptional profile, as determined by RNA-sequencing. (**A**) Oncogenic and tumor suppressor transcription factors measured by single gene set enrichment analysis (ssGSEA). (**B**) Expression of target genes of FOXM1 evaluated by RNA-sequencing. (**C)** Expression of Wnt signaling genes and β-catenin/TCF complex related genes evaluated by RNA-sequencing. (**D**) Heat map showing levels of expression of stemness- and invasiveness-associated genes. (**E**) Set of genes downregulated after treatment with DFS for 72 h. (**F**) Heat map showing levels of expression of cell cycle-related genes.

RNA sequencing was performed to assess the effects of DFS treatment on the transcriptional reprogramming of GBM TSs. GBM TSs used in the RNA sequencing were treated with 10 µM of DFS for 72 h, and the experimental results showed no difference in the effect of DFS between the two GBM TSs, TS13-64 and TS15-88. Samples were subjected to single sample gene set enrichment analysis (ssGSEA) using transcription factor target gene sets^23^. DFS significantly reduced the activities of several transcription factors associated with tumorigenesis, stemness, invasiveness, and mesenchymal transition, while significantly increasing the activities of tumor suppressor transcription factors (Fig. 4A). And DFS reduced expression level of mRNAs associated with the FOXM1 target genes (Fig. 4B). It also consistently downregulated the levels of expression of mRNAs associated with the Wnt signaling pathway and with the activation of β-catenin/TCF complex-associated genes (Fig. 4C), as well as stemness- and invasiveness-related genes (Fig. 4D). Analysis of differentially expressed genes (DEGs) by DFS-treated and control GBM TSs showed that the DEGs significantly downregulated in DFS treated cells included several cell cycle-associated gene sets (Fig. 4E), and we also confirmed that the expression of most genes involved in sub-G1, S, and G2 phase transitions were downregulated (Fig. 4F). With these results, DFS is considered to induce cell cycle arrest.

### Therapeutic responses in a mouse orthotopic xenograft model

**Figure 5:**
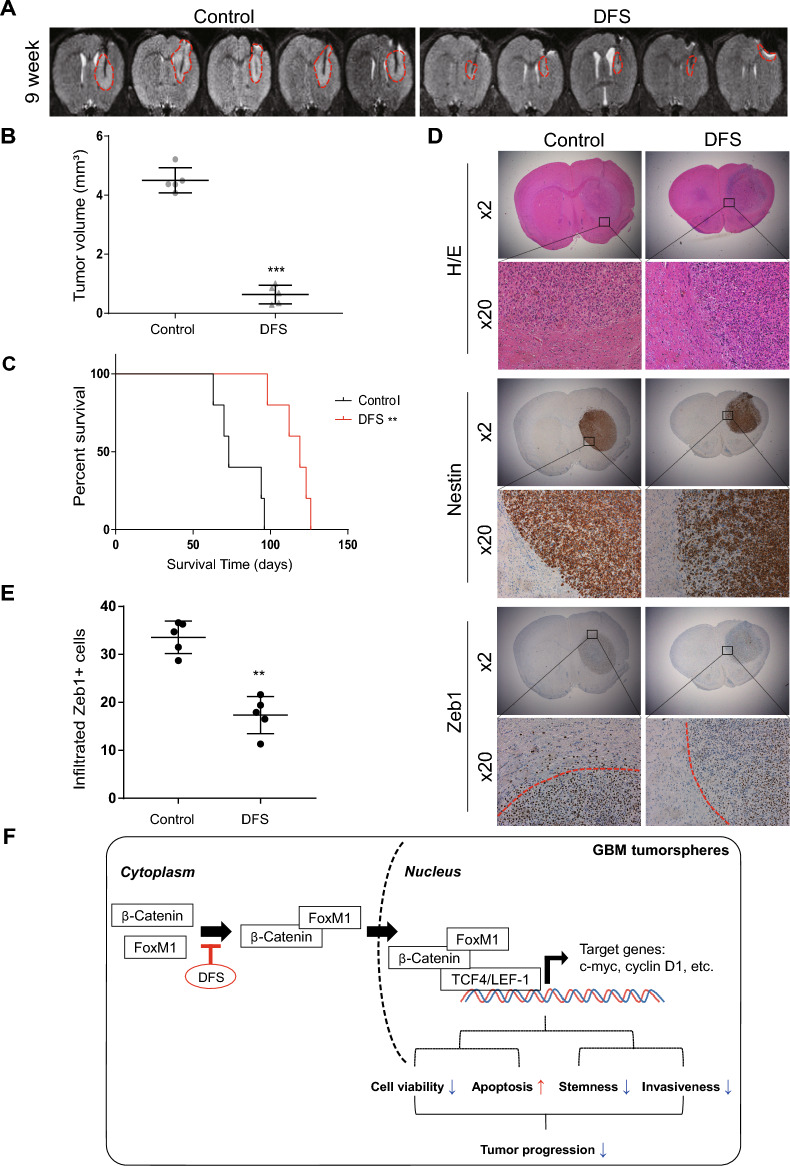
Therapeutic effects of DFS in orthotopic xenograft models. (**A**,**B**) Tumor volumes of orthotopic xenograft models measured by magnetic resonance imaging (MRI). (**C**) Kaplan–Meier analysis of survival probability of each group of mice, with comparisons by log-rank tests (P < 0.05) with Bonferroni adjustment. (**D**,**E**) To identify invading cells, section of the brains of killed mice were immunostained for Zeb1. From 10 photos captured for each mouse, the number of infiltrating Zeb+ cells (outside the red line in (**D**)) was tallied for each group (means ± SEM; *P < 0.01 compared with control). (**F**) Schematic summary of the study.

The effects of DFS pretreatment of GBM TSs on tumor growth were evaluated in orthotopic xenograft models of GBM using TS13-64. Magnetic resonance imaging (MRI) showed that tumors in the control group were larger than those in the DFS-pretreated group, indicating that pretreatment with DFS reduced the ability of GBM TSs to promote tumor growth in a mouse orthotopic xenograft model (Fig. 5A,B). Kaplan–Meier survival analysis showed that DFS pretreatment significantly prolonged mouse survival compared with the control group (p = 0.0018) (Fig. 5C). Collectively, these results clearly demonstrate that DFS was able to reduce the tumorigenesis capacity of TS13-64.

In addition, immunohistochemistry (IHC) was performed on mouse brain tissue that had been tested for stemness and invasiveness of GBM TSs to determine how DFS affects local microenvironment cells (Fig. 5D). In DFS treated TS, expression of Nestin, the marker of stemness, was downregulated and tumor edges were soother than TS untreated group. The amount of Zeb1-positive cells, which indicate invading cells, was also reduced outside the gross tumor mass by DFS (Fig. 5E).

## Discussion

GBM is the most common and malignant primary brain tumor in adults with an unfavorable prognosis^3^. It exhibits malignant progression, resistance to conventional treatment, and infiltrative features^7^. Numerous active researches are being conducted on the origin of GBM, from neural stem cells in the subventricular zone of the adult human brain, and its treatment methods^4^. Although multimodal therapeutic strategy has been suggested including surgery, chemotherapy, and radiotherapy, the overall survival of patients with GBM is still very low at 14.6 to 21.1^2^. This study was designed to find a new treatment method for this challenging disease.

DFS, a lignan isolated from a methanol extract of stems of *A. japonica*^24^. *A. japonica* is a plant widely used as both a food and a medicinal herb in east Asia^19,24^. It is effective in various medical conditions, such as fever, diarrhea and hemorrhage, because it contains diverse pharmacologically active components., In previous study, DFS interfered with β-catenin mediated genes through lysosomal-dependent degradation of FOXM1 protein in colon cancer^19^. However, there has been no study on whether DFS is effective in GBM. The present study hypothesized that DFS would exert a proliferation-inhibiting effect in GBM because of the fact that stemness and tumorigenicity are regulated by FOXM1/β-catenin interactions in glioma stem cells^18^.

According to previous study, GBM TS is a prominent phenotype with features such as stemness and invasiveness, and it has been found to have value in clinical use^6^. In this study, GBM cells were isolated from a specimen derived from a patient diagnosed with GBM, and then TS was obtained through cell culture. Among the GBM TSs used in this experiment, the results were analyzed for two GBM TSs, denoted TS15-88 and TS13-64, which showed a clear response after DFS treatment. GBM TS itself can be considered to show the same treatment response as in the patient’s GBM tissue, and has the advantage of being suitable for applying manipulations for in vivo experiments by implantation in a mouse model.

The Wnt/β-catenin signaling pathway is aberrantly activated during the development and progression of various human cancers. This pathway plays important roles in the tumorigenesis and progression of malignant astrocytic gliomas^25–28^, including in the promotion of stem cell genes^29–32^, in tumor migration and invasion, and in the epithelial-to-mesenchymal transition (EMT)^33–35^. FOXM1, a key mediator of the Wnt/β-catenin pathway, is overexpressed in many human cancers, including glioma. The finding in the present study, that the level of FOXM1 mRNA was significantly higher in GBM than in normal brain tissue, suggests that targeting FOXM1 in GBM can suppress tumor progression. And this result suggests the possibility that DFS may also have a therapeutic effect on GBM, considering the mechanism that DFS exerts anti-cancer effects by acting on FOXM1.

The interaction between FOXM1 and β-catenin has been shown to promote the nuclear translocation of β-catenin and to increase the expression of β-catenin target genes, thereby promoting self-renewal and tumorigenesis of GBM initiating cells (GICs)^18^. The present study found that DFS reduced interactions between FOXM1 and β-catenin in the nucleus, subsequently reducing the expression of proteins such as cyclin D1 and cMyc by suppressing formation of the complex with TCF4^19,24^. Moreover, the levels of expression of mRNA associated with Wnt signaling pathway and the activation of β-catenin/TCF complex-related genes were downregulated in DFS treated GBM TSs. The fact that DFS inhibited the FOXM1/β-catenin interaction and subsequently suppressed the expression of β-catenin/TCF complex-related genes in GBM TSs suggests that DFS, as a consequence, can regulate TCF in transcription level. It is known that FOXM1 regulate transcription by the direct binding of small molecules such as FDI-6. And DFS-induced FOXM1 protein degradation is considered to be dependent on lysosome^19^. We confirmed that DFS significantly reduced the enrichment score of FOXM1 for oncogenic transcription factors which shows that the expression of target genes of FOXM1 were reduced overall as shown in Fig. 4A.

According to previous studies, nuclear β-catenin is highly correlated with TCF, a DNA-binding transcription factor^18,19^. In this study, we investigated the fraction of β-catenin in both the cytosol and nucleus to quantitatively confirm the subcellular localization of β-catenin (Fig. 3E). As a result, we confirmed that the expression of active β-catenin was decreased in the nucleus. In addition, it was confirmed that FOXM1/β-catenin binding, a factor regulating the downstream of Wnt signaling and the transcription function of β-catenin, was weakened by DFS, and consequently, reduced β-catenin in the nucleus.

The present study investigated the mechanisms of action of DFS in GBM TSs through the FOXM1/β-catenin signaling pathways. Inhibition of FOXM1 was found to reduce cell proliferation and induce apoptosis^36,37^, whereas overexpression of FOXM1 was found to promote cell cycle progression^38,39^ and cell proliferation by directly activating the transcription of genes encoding cyclin D1 and cyclin B1^40,41^. The present study found that DFS inhibited the viability of GBM TSs and significantly increased the number of cells in sub-G1 phase of the cell cycle, indicating that DFS cytotoxicity is mediated by apoptosis. In addition, DFS decreased the protein expression of Bcl-2 and increased the expression of cleaved caspase-3 and BAX. Analysis of DEGs showed that DFS upregulated genes involving the sub-G1, S and G2 phase transitions of the cell cycle. Collectively, these results demonstrated that DFS has anticancer potential in human GBM cells through the induction of cell cycle arrest and apoptosis.

FOXM1 has been shown to promote the stemness of GBMs by regulating the master stem cell regulator SOX2^42^. Furthermore, FOXM1 promotes EMT and is deeply involved in the acquisition in pancreatic cancer cells of a cancer stem cell (CSC) phenotype^43^. The present study investigated the prominent phenotypes of GBM TSs, which have stemness and invasiveness. Stem-like cells that induce GBM TS in tumors are known to affect treatment resistance and recurrence, making the inhibition of stemness and invasiveness a positive indicator for evaluating the potential efficacy of DFS.

We used two TSs, TS13-64 and TS15-88, for all other experiments except in in vivo mouse model which was performed only with TS13-64 since there was no significant difference in the effect of DFS between these two TSs. And TS13-64 had shorter mouse survival time and more overall experiment data was available compared to TS15-88. Moreover, unfortunately, in vivo experiments using a orthotopic xenograft mouse model were performed only as a pretreatment method, not in other methods such as intravenous, intraperitoneal, or local injection. We previously conducted an experiment to administer DFS to mice by intraperitoneal injection, however, the effect of DFS from the experiment was not seen. In addition, because DFS is a new drug that is still in the research stage, the mechanism of action in the brain has not been established. So we conducted an experiment using the pretreatment technique with reference to other literatures^48,49^. In order to overcome this limitation, research on injection methods other than pretreatment should be conducted in the next stage of DFS study which increase the utilization of this drug.

In summary, DFS can interfere with signaling steps of stemness, invasiveness and proliferation by suppression of FOXM1 (Fig. 5F). DFS consequently inhibited the proliferation of GBM TSs in vivo, prolonging the survival time of mice with orthotopic GBM xenografts. These results were obtained from GBM patient-derived cells and a corresponding mouse model, highlighting the clinical applicability of DFS as a new treatment for GBM. DFS may be a potential therapeutic agent to inhibit GBM progression by increasing apoptosis and reducing cell viability, stemness and invasiveness in patients with refractory GBM. This study is meaningful in that it is the first study to confirm whether DFS also exhibits an anti-cancer effect in GBM, based on the fact that it exhibits an inhibition effect in colon cancer. There are many studies dealing with medical treatment of GBM, but there are few studies that suggest new therapeutic agents other than those currently used. This is a study that confirmed the possibility that DFS could be used as another option for medical treatment of GBM in addition to drugs such as temozolomide and bevacizumab. In the future, follow-up studies should be conducted to confirm whether DFS is suitable for clinical use as a treatment for patient with GBM. It is expected that DFS will be a key material that can take one step closer to conquering the GBM.

## Materials and methods

### Culture of GBM TSs and reagents

GBM TSs were derived from two individual patients with GBM. These GBM TSs, denoted TS15-88 and TS13-64, were established from fresh GBM tissue specimens, as approved by the institutional review board of the Yonsei University College of Medicine. TSs were cultured according to standard methods^44–46^. Briefly, cells were cultured in TS complete medium, consisting of Dulbecco’s modified Eagle’s medium/F12 (Mediatech, Manassas, VA, USA), 1 × B27 (Invitrogen, Carlsbad, CA, USA), 20 ng/mL basic fibroblast growth factor (bFGF; Novoprotein, Summit, NJ, USA) and 20 ng/mL epidermal growth factor (EGF; Novoprotein). All in vitro experiments were performed under the culture conditions for TSs. DFS, an anti-GBM lignan, was isolated from a chloroform soluble fraction of *A. japonica* extracts, as described^19^.

### Western blot

Lysates of GBM TSs were separated by sodium dodecyl sulfate-polyacrylamide gel electrophoresis (SDS-PAGE) on Tris–glycine gels. Cytosolic and nuclear fractions were prepared using cytoplasmic and nuclear extraction reagents (Thermo Scientific, Rockford, IL, USA), respectively, according to the manufacturer’s instructions. Proteins were transferred to nitrocellulose membranes and probed with antibodies against cleaved capase-3, CD133, PDPN, β-catenin, active-β-catenin, CD44, snail, Cyclin D1 and cMYC (Cell Signaling Technology, Danvers, MA, USA); Sox2 (Merck Millipore, Darmstadt, Germany); Msi-1 (Abcam, Cambridge, UK); nestin (Novus Biologicals, Centennial, CO, USA); N-cadherin, Zeb1 (Sigma-Aldrich, St. Louis, MO, USA); Twist, Oct3/4, BAX, Bcl-2, PARP and GAPDH (Santa Cruz Biotechnology, Dallas, TX, USA); and FOXM1 (Bethyl Laboratories, Montgomery, TX, USA), along with Western Lightning Plus-enhanced chemiluminescence reagent (PerkinElmer, Lawrence, MA, USA). Images were captured using an ImageQuant LAS 4000 mini (GE Healthcare Life Sciences, Little Chalfont, UK). We expressed all the results of Western blot as a violin plot, and added this as a Supplementary 5. The original image of full-length blot was added as a Supplementary 6.

### Immunoprecipitation

To determine interactions between FOXM1 and β-catenin, GBM TSs were treated with DFS for 72 h, lysed with lysis buffer and incubated with anti-FOXM1 or anti-β-catenin antibody on a rotator overnight at 4 °C. Protein-antibody-protein A/G agarose complexes were prepared using protein A/G sepharose beads (Santa Cruz Biotechnology) for 4 h at 4 °C. After extensive washing with PBST, the immunoprecipitated complexes were eluted with 0.1 M glycine–HCl (pH 2.7), and neutralized with neutralizing buffer (1 M Tris–HCl, pH 9.0). The precipitates were then resuspended with SDS sample buffer and boiled for 10 min. The precipitated proteins were subjected to immunoblotting with the indicated antibodies.

### GST pull-down assay

GST-tagged β-catenin was expressed in DH5α E. coli strain and purified by affinity chromatography on glutathione-Sepharose 4B beads (GE Healthcare). Flag-tagged FOXM1 was prepared by lysate by transfecting according to manufacturer's recommendation in TS15-88 and TS13-64, respectively. Confirming the purity of recombinant proteins was done with Coomassie brilliant blue staining by gel separation. For GST-pulldown assay, purified recombinant GST-β-catenin and GST control protein were co-incubated with 20 μl of 50% glutathione agarose beads slurry (GE Healthcare) in binding buffer at about 1 h at 4 °C. The bead elution process was done after washing the beads 3 times with a binding buffer. Thereafter, SDS sample buffer heating was assessed and immunoblotting was done with anti-Flag antibody.

### Luciferase reporter assay

Canonical TCF/LEF signaling activity was measured using TOPflash/FOPflash reporter assays. Briefly, 1 × 10^5^ cells were seeded into each well of a 24-well plate for 1 day before transfection. The cells were transfected with 100 ng of firefly luciferase reporter plasmid and 10 ng of internal control plasmid pRL-TK (Promega, Madison, WI, USA) using Lipofectamine 3000 (Invitrogen) for 72 h according to the manufacturer's protocol. Samples were lysed with 1 × reporter lysis buffer for 15 min and centrifuged, and the supernatants were used for luciferase and β-galactosidase assays, according to the manufacturers' protocols. After confirming that the cells had been transfected using β-galactosidase assays, luciferase activity in 10 μg of protein lysate was quantified using the Dual Luciferase Reporter Assay System (Promega). Each sample was analyzed in triplicate.

### Evaluation of cell viability

The effects of DFS on the survival of GBM TSs were determined using WST assays (Promega). Briefly, 1 × 10^4^ single dissociated GBM TSs were seeded into each well of a 96-well plate. The plates were incubated at 37 °C for 24 h and treated with DFS for 72 h. WST reagent (10 μl/well) was added and absorbance was measured at 450 nm after incubating at 37 °C for 1 h. Each experiment was repeated three times in triplicate, and the results are expressed as the percentage of viable cells relative to controls.

### Evaluation of ATP levels

Dispersed GBM TSs were plated in 96-well plates at a density of 1 × 10^4^ cells per well. After incubating for 24 h, TSs were treated with DFS for 3 days, and then ATP levels were compared using the CellTiter-Glo Luminescent Cell Viability Assay (Promega) according to the manufacturer’s protocol. Briefly, a volume of CellTiter-Glo Reagent equal to the volume of cell culture medium was added to each well, after which cells were incubated at room temperature for 10 min, and luminescence was measured.

### Cell cycle and apoptosis analysis

To analyze the cell cycle, GBM TSs treated with DFS for 72 h were collected, fixed with 70% ethanol, dissociated with accutase (Sigma), and stained with 100 µg/ml of propidium iodide (PI, Sigma) at 4 °C for 15 min in the dark. To evaluate DFS-induced apoptosis in GBM TSs, GBM TSs were treated with the indicated concentration of DFS. Apoptotic cell death was detected by double staining with fluorescein-isothiocyanate (FITC)-conjugated annexin V (BioLegend, San Diego, CA, USA) and PI. The stained cells were analyzed using an LSR II flow cytometer (BD Biosciences, Bedford MA, USA).

### Sphere formation assays

Ten single dissociated GBM TSs were seeded in each well of a 96-well plate and treated with DFS after 24 h. The plates were incubated for 3 weeks in TS complete medium, with medium changed every week. The number of sphere-positive wells was counted, and the ratio of sphere-positive wells in the treated relative to the control group was calculated. The radius of spheres in each group was also measured. Images were captured and analyzed using ToupView software (ToupTek Photonics, Zhejiang, China).

### Three-dimensional (3D) invasion assays

Each well of a 96-well plate was filled with mixed matrix composed of matrigel (Corning Incorporated, Tewsbury, MA, USA), collagen type I (Corning Incorporated) and TS complete medium. Single spheroids had been seeded inside the matrix prior to gelation, followed by the addition of TS complete medium over the gelled matrix to prevent drying. The invaded area was quantified as occupied area at (72 h − 0 h)/0 h.

### Immunofluorescence

GBM TSs were incubated by attaching them to coverslips in 24-well plates. After treatment for 48 h with or without treatment, cells were fixed with 3.8% formaldehyde for 10 min. The permeabilization process were done with 0.1% NP40/PBS and blocked with 1% BSA/PBS for 1 h. To simultaneously observe the subcellular localization of β-catenin and FOXM1, β–catenin (1:1000) and FOXM1 (1:1000) were incubated overnight at 4 °C. Cells then washed with PBS, and incubated with a secondary antibody conjugated with Alexa Fluor 488, 546 (1:1000) at room temperature for 1 h. Cells then washed with PBS and mounted with a mounting solution containing DAPI (Vetcached H-1200) and imaged with a confocal microscope (LSM700; Carl Zeiss MicroImaging, Inc.). Acquired cell images were analyzed using ZEN 2009 software. The ImageJ software was used to calculate the relative fluorescence intensity of FOXM1 and β-catenin.

### Processing of transcriptome data

Total RNA was extracted from GBM TSs and used for RNA sequencing. The quality of the reads was checked using FastQC (v.0.10.1) and the sequencing adapters were removed using Trimmomatic (v. 0.38). Low quality reads were filtered according to the following criteria: reads containing more than 10% of skipped bases (marked as ‘N’s), reads containing more than 40% of bases with quality scores less than 20, and reads whose average quality scores of each read were less than 20. Filtered reads were mapped to the human reference genome using the aligner Tophat^47^. Gene expression levels were measured with Cufflinks v2.1.1^48^, using the gene annotation database of Ensembl release 72^49^. Non-coding regions were removed using the –mask option. Using GENE-E software, the average linkage of hierarchical clustering was determined with Pearson’s correction as a distance metric and expression levels depicted as heat maps.

### Mouse orthotopic xenograft model

Male athymic nude mice aged 4–8 weeks (Central Lab. Animal Inc., Seoul, Korea) were housed in micro-isolator cages under sterile conditions and monitored for at least one week before study initiation to ensure proper health. Lighting, temperature, and humidity were controlled centrally. Dissociated Luc-TS13-64 cells were pretreated with 10 µM of DFS for 72 h using the pretreatment protocol^50,51^. Cells were selected by trypan blue staining, and 5 × 10^5^ viable cells were implanted into the right frontal lobe of each mouse at a depth of 4.5 mm using a guide-screw system^44–46,52^. Mice showing a > 15% reduction in body weight compared with the maximum were euthanized according to the approved protocol. For immunohistochemistry, 4-μm-thick sections were obtained with a microtome and transferred onto adhesive slides. Antigen retrieval and antibody attachment were performed using an automated instrument (Discovery XT, Ventana Medical Systems). Nestin and Zeb1 were detected using a peroxidase/3,3ʹ-diaminobenzidine staining system.

### Statistical analysis

Levels of significance for comparisons among treatment groups were determined using one-way ANOVA with Tukey’s post hoc test for multiple comparisons. Survival was analyzed using the Kaplan–Meier method and compared by log-rank tests. All the graphs and statistical analyses were performed using GraphPad Prism 9 (GraphPad Software Inc., San Diego, CA, USA) software, with P-values < 0.05 considered statistically significant.

### Ethical approval and consent to participate

All patients provided written informed consent, and permission for specimen sampling and evaluation was obtained from the Institutional Review Boards at our institutes (no. 4-2021-1319). This study was carried out in accordance with relevant guidelines and ethical regulations of the institutional and national research committee.

All applicable international, national, and/or institutional guidelines for the care and use of animals were followed. All procedures involving animals followed the ethical standards of the institution or practice at which the studies were conducted. All procedures related with the in vivo experiments and animal care were approved by the Committee for the Care and Use of Laboratory Animals at Yonsei University College of Medicine (no. 2020-0248) and were conducted according to the guidelines of the Care and Use of Laboratory Animals published by the US National Institutes of Health. We complied with the ARRIVE guideline for this reporting of animal experiment conducted in the present study.

This article does not contain any studies involving human participants performed by any of the authors.

### Consent to publish

The work described here has not been published before and is not under consideration for publication elsewhere. All authors have approved the manuscript.

## Supplementary Information

Supplementary Figures.

## Data Availability

The datasets generated and/or analyzed during the current study are not publicly available due to the risk of compromising individual privacy but are available from the corresponding author on reasonable request and with an appropriate agreed upon collaboration.
