# Author Correction: A lignan from *Alnus japonica* inhibits glioblastoma tumorspheres by suppression of FOXM1

**DOI:** 10.1038/s41598-025-02265-8

**Published:** 2025-05-30

**Authors:** Jin‑Kyoung Shim, Seung Hoon Lim, Ji Hye Jeong, Ran Joo Choi, Yoojung Oh, Junseong Park, Sunghee Choi, Junpyo Hong, Seo Jin Kim, Ju Hyung Moon, Eui Hyun Kim, Wan‑Yee Teo, Bong Jin Park, Jong Hee Chang, Jae‑Ha Ryu, Seok‑Gu Kang

**Affiliations:** 1 Department of Neurosurgery, Brain Tumor Center, Severance Hospital, Yonsei University College of Medicine, Seoul, Republic of Korea; 2 Department of Neurosurgery, Kyung Hee University College of Medicine, Seoul, Republic of Korea; 3 Research Institute of Pharmaceutical Sciences and College of Pharmacy, Sookmyung Women’s University, Seoul, Republic of Korea; 4 Precision Medicine Research Center, College of Medicine, The Catholic University of Korea, Seoul, Republic of Korea; 5 Cancer and Stem Cell Biology Program, Duke-NUS Medical School, Singapore, Singapore; 6 Institute of Molecular and Cell Biology, A*STAR, Singapore, Singapore; 7 Department of Medical Science, Yonsei University Graduate School, Seoul, Republic of Korea

Correction to: *Scientific Reports* 10.1038/s41598-022-18185-w, published online 17 August 2022

The original version of this Article contained errors.

As a result of an error during assembly of Figure 2, the GAPDH control corresponding to experiments shown in Figure 2B was also used in Figure 2D. Western blots experiments were originally performed in triplicate; Figure 2D is now corrected in its entirety with a data derived from an independent replicate. The original version of Figure 2 is shown below.

Additionally, as a result of another error during figure assembly in Figure 3D a control panel for TS13-64 is a duplication of a control panel for TS15-88. This figure is now updated to show the correct control data for TS13-64. The original version of Figure 3 is shown below.

The Article has been updated.

**Fig. 2:**
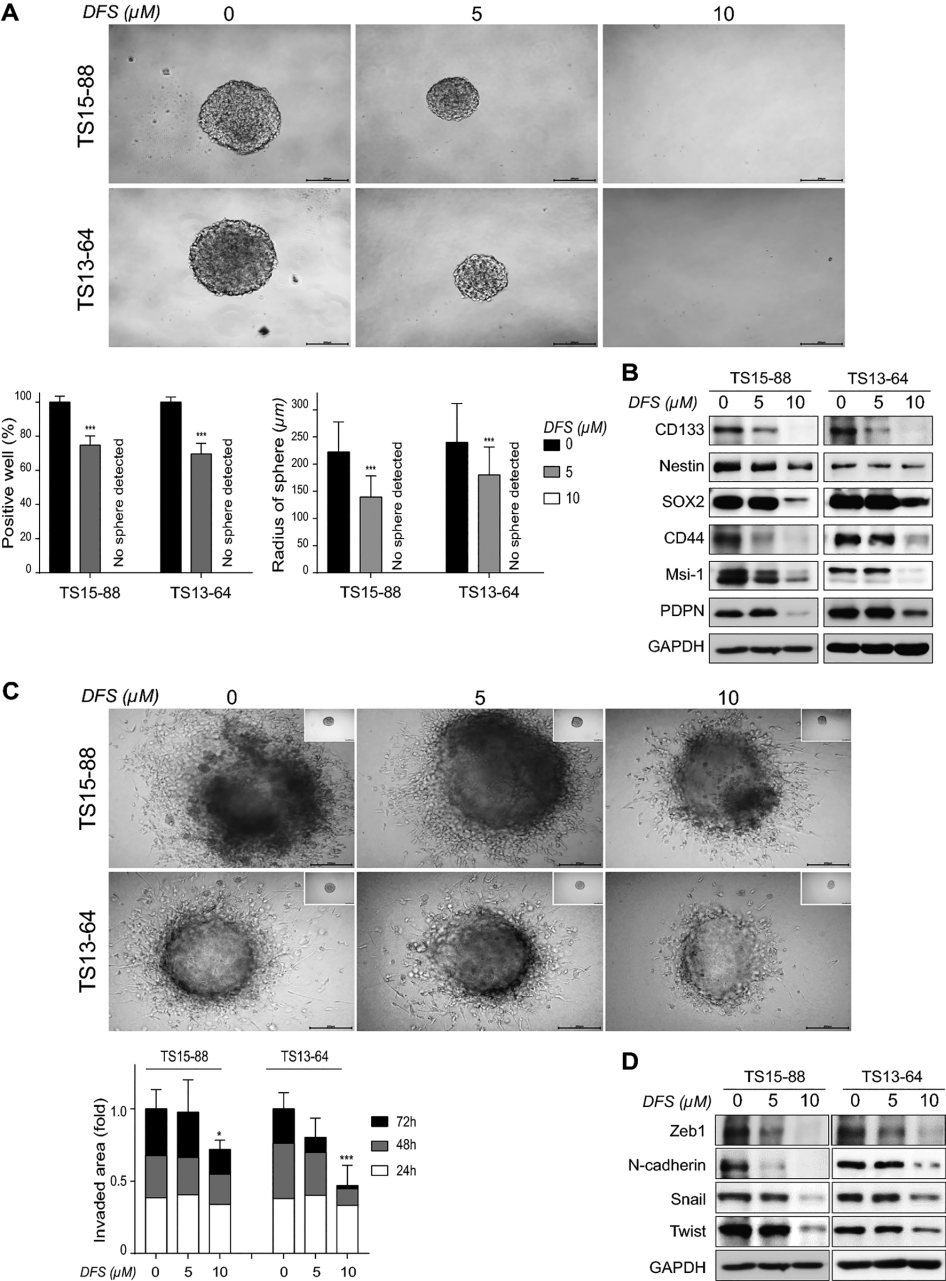
Inhibitory effects of DFS on stemness and invasiveness of GBM TSs. (**A**) Stemness was determined by Sphere formation assay. Cells were treated with DFS for 3 weeks and the percentages of sphere-positive wells and sphere radii were calculated. (**B**) GBM TSs were treated with DFS for 72 h and stemness-associated protein levels were determined by western blotting. (**C**) GBM TSs were cultured on matrigel/collagen matrix with DFS for 72 h, and invasiveness was quantified by measuring areas of migration. (**D**) Western blotting assays of the expression of EMT-related proteins. All Images are × 10 original magnification (scale bar = 200 μm). Differences among groups were compared by one-way ANOVA with Tukey’s post hoc test; means ± SD; *P < 0.05, **P < 0.01, ***P < 0.001, between indicated groups or compared with controls.

**Fig. 3:**
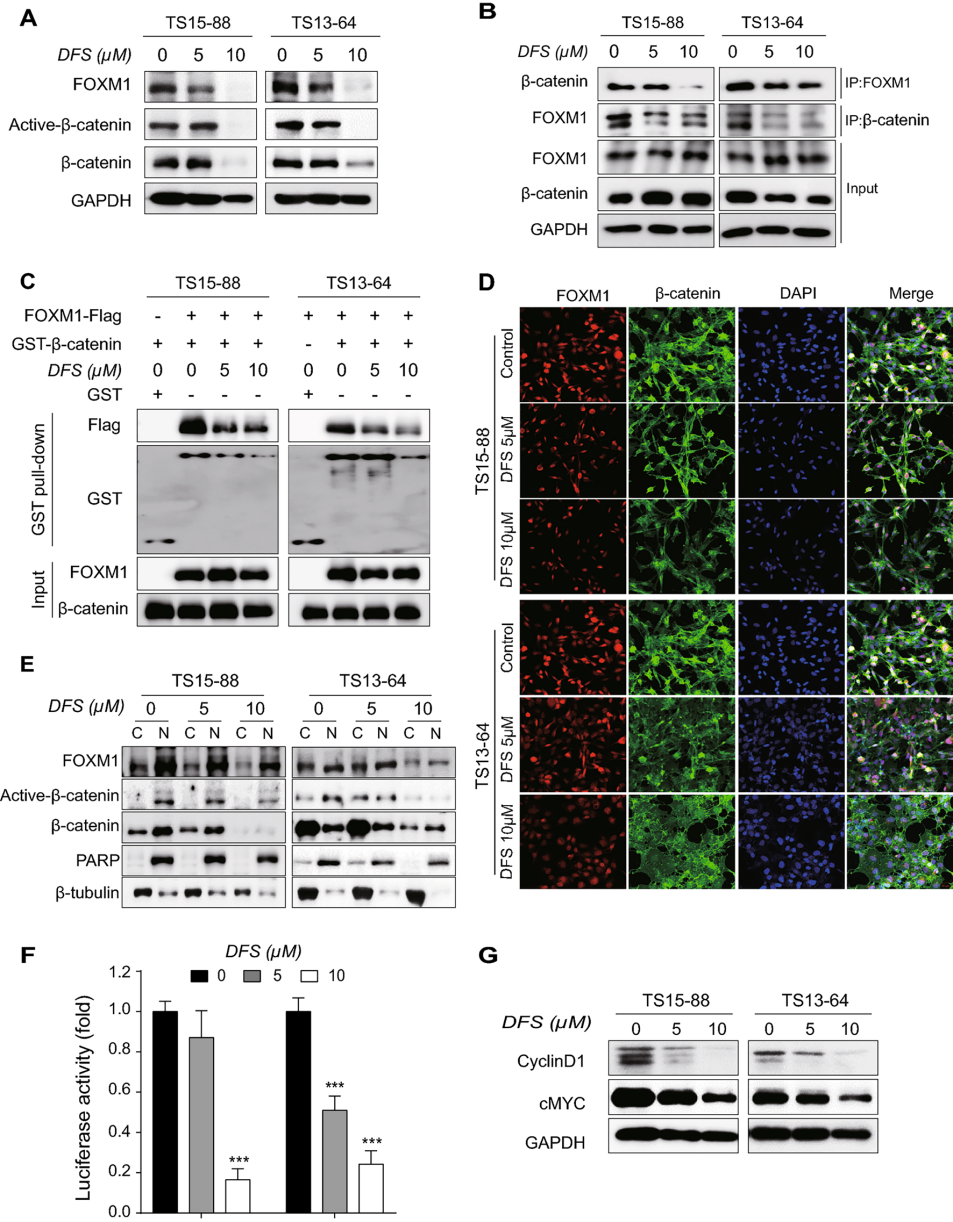
Inhibition of FOXM1/ β-catenin interaction by DFS. (**A**) GBM TSs were treated with DFS for 72 h and the protein levels of FOXM1, active-β-catenin and total β-catenin were determined by western blotting. (**B**) DFS-treated GBM TSs were immunoprecipitated with anti-FOXM1 and β-catenin antibody, and endogenous expression of FOXM1 and β-catenin proteins were determined by western blotting to determine interactions between them. (**C**) GST pull-down assay to confirm whether FOXM1 and β-catenin interactions are direct or indirect. (**D**) Immunofluorescence to determine whether DFS affects the nuclear translocation of β-catenin (magnification × 20). (**E**) Levels of FOXM1 and β-catenin proteins in the cytosolic and nuclear fractions of GBM TSs determined by western blotting. The cytosolic and nuclear fractions are denoted by C and N, respectively. Cytosolic marker: β-tubulin; nuclear marker: PARP. (**F**) Measurements of β-catenin/TCF signaling activity in GBM TSs transfected with TOPflash or FOPflash luciferase vector. (**G**) Expression of proteins encoded by β-catenin downstream genes (cyclin D1, cMYC) measured by western blotting. Differences among groups were compared by one-way ANOVA with Tukey’s post hoc test; means ± SD; *P < 0.05, **P < 0.01, ***P < 0.001, between indicated groups or compared with controls.

