# Supplementary material for: A lignan from Alnus japonica inhibits glioblastoma tumorspheres by suppression of FOXM1
Source: Sci Rep. 2022 Aug 17;12:13990. doi: 10.1038/s41598-022-18185-w (PMC9385634; doi:10.1038/s41598-022-18185-w)
Supplement: Supplementary file 1 — Supplementary Figures. [file 41598_2022_18185_MOESM1_ESM.docx]

**A lignan from *Alnus japonica* inhibits glioblastoma tumorspheres by suppression of FOXM1**

Jin-Kyoung Shim^1,#^, Seung Hoon Lim^1,2#^, Ji Hye Jeong^3^, Ran Joo Choi^1^, Yoojung Oh^1^, Junseong Park^1,4^, Sunghee Choi^3^, Junpyo Hong^1^, Seo Jin Kim^1^, Ju Hyung Moon^1^, Eui Hyun Kim^1^, Wan-Yee Teo^5,6^, Bong Jin Park^2^, Jong Hee Chang^1^, Jae-Ha Ryu^3^, Seok-Gu Kang^1,7^

^1^Department of Neurosurgery, Brain Tumor Center, Severance Hospital, Yonsei University College of Medicine, Seoul, Republic of Korea; ^2^Department of Neurosurgery, Kyung Hee University College of Medicine, Seoul, Republic of Korea; ^3^Research Institute of Pharmaceutical Sciences and College of Pharmacy, Sookmyung Women’s University, Seoul, Republic of Korea; ^4^Precision Medicine Research Center, College of Medicine, The Catholic University of Korea, Seoul, Republic of Korea; ^5^Cancer and Stem Cell Biology Program, Duke-NUS Medical School, Singapore; ^6^Institute of Molecular and Cell Biology, A*STAR, Singapore; ^7^Department of Medical Science, Yonsei University Graduate School, Seoul, Republic of Korea

^#^ These authors contributed equally to this work.

*Corresponding Author

Seok-Gu Kang, M.D., Ph.D., Department of Neurosurgery, Brain Tumor Center, Severance Hospital, Yonsei University College of Medicine, 50-1 Yonsei-ro, Seodaemun-gu, Seoul 03722, Republic of Korea. Tel: +82-2-2228-0882; Fax: +82-2-393-9979; E-mail: seokgu9@gmail.com

Jae-Ha Ryu, Ph.D., College of Pharmacy, Sookmyung Women’s University, 100 Cheongpa-ro 47 Gil, Yongsan-gu, Seoul 04310, Republic of Korea. Tel: +82-2-710-9568; Fax: +82-2-2077-7322; E-mail: ryuha@sookmyung.ac.kr

Figure S1. Surface Plasmon Resonance (SPR) analysis. SPR analysis was performed using the BIAcore 2000 model (GE Healthcare) at room temperature with running buffer (10mM HEPES, 0.15M NaCl, 0.005% Tween-20) containing 1% dimethyl sulfoxide (DMSO). The pH scouting for β-catenin (Addgene) immobilization was performed in 5 mM acetate buffer at pH 4.0. β-catenin was immobilized on a CMD 200M sensor chip to 2,000 and 1,000 response units (RU) with standard amine coupling at pH 4. FOXM1(Addgene, 0.4µg/ml) was injected into the flow cells in the absence or presence of DFS (12.5-50µM) with a flow rate of 10 µl/min for 180 sec and allowed to dissociate for 150 sec. The KD values were obtained using the fitting tool of the BIAevaluation software (Biacore).


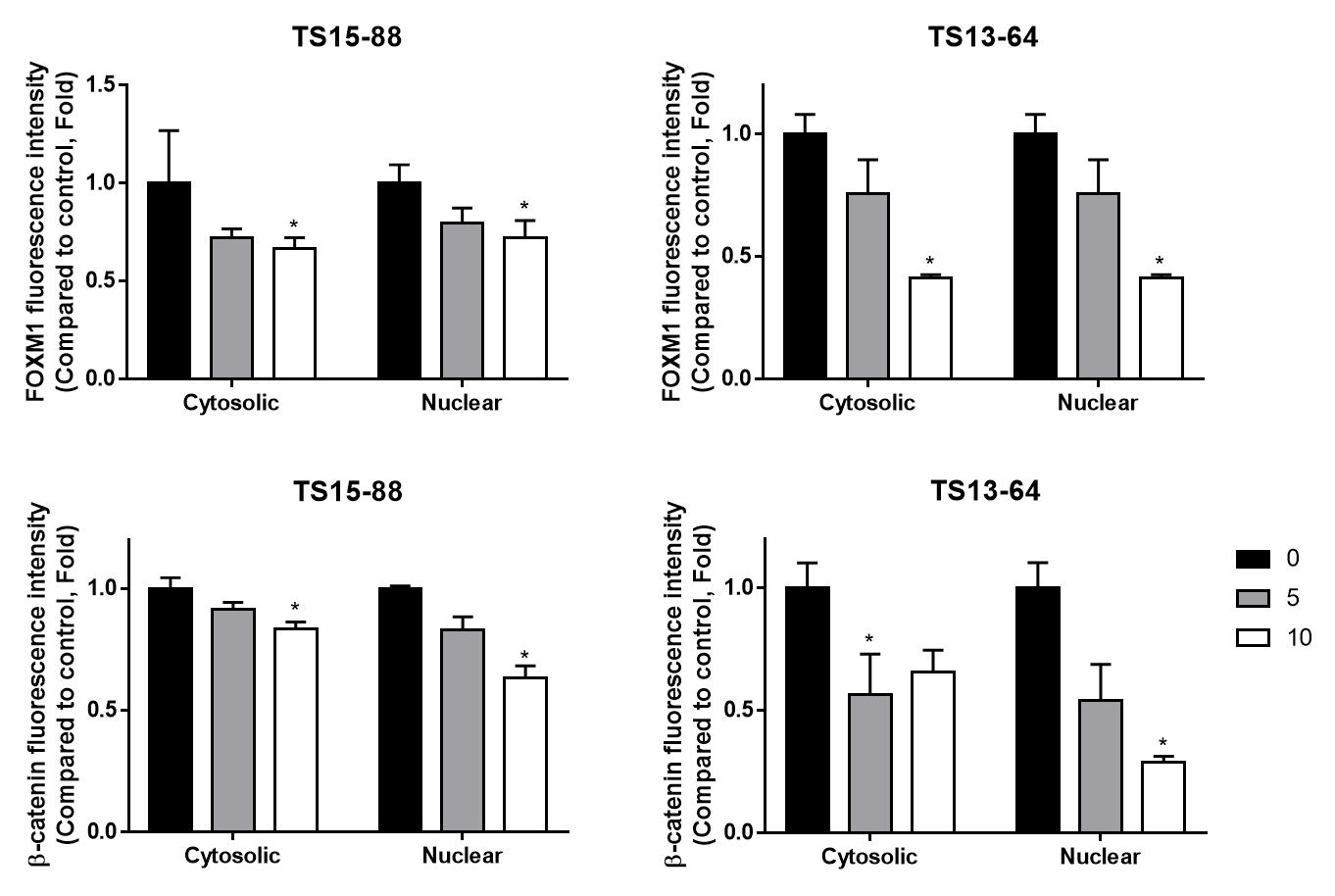


Figure S2a. Immunofluorescence intensity quantification of FOXM1 and β-catenin.

Figure S2b. Immunofluorescence intensity quantification of nuclear:cytoplasmic ratios of β-catenin.


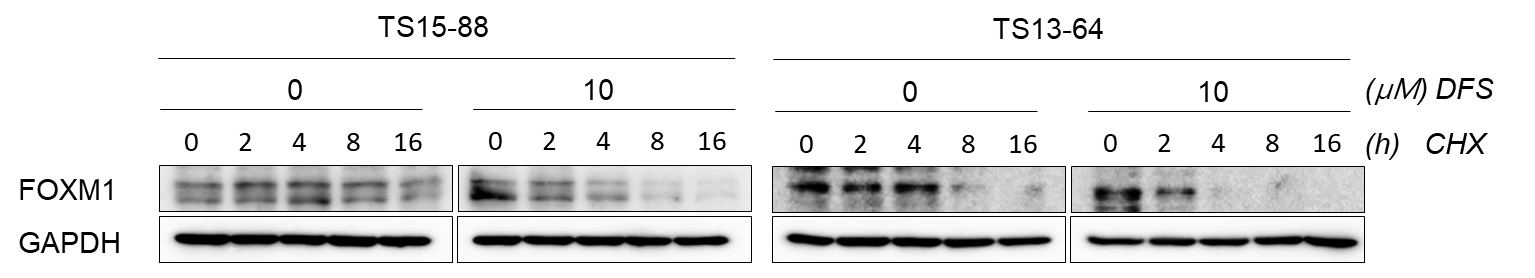


Figure S3. GBM TSs were treated with 10 µM DFS for 24 hours before being treated with 10 µg/ml cycloheximide (CHX) to inhibit protein synthesis. Cellular proteins were extracted at various periods after CHX administration, and the level of FOXM1 protein was determined by western blotting.


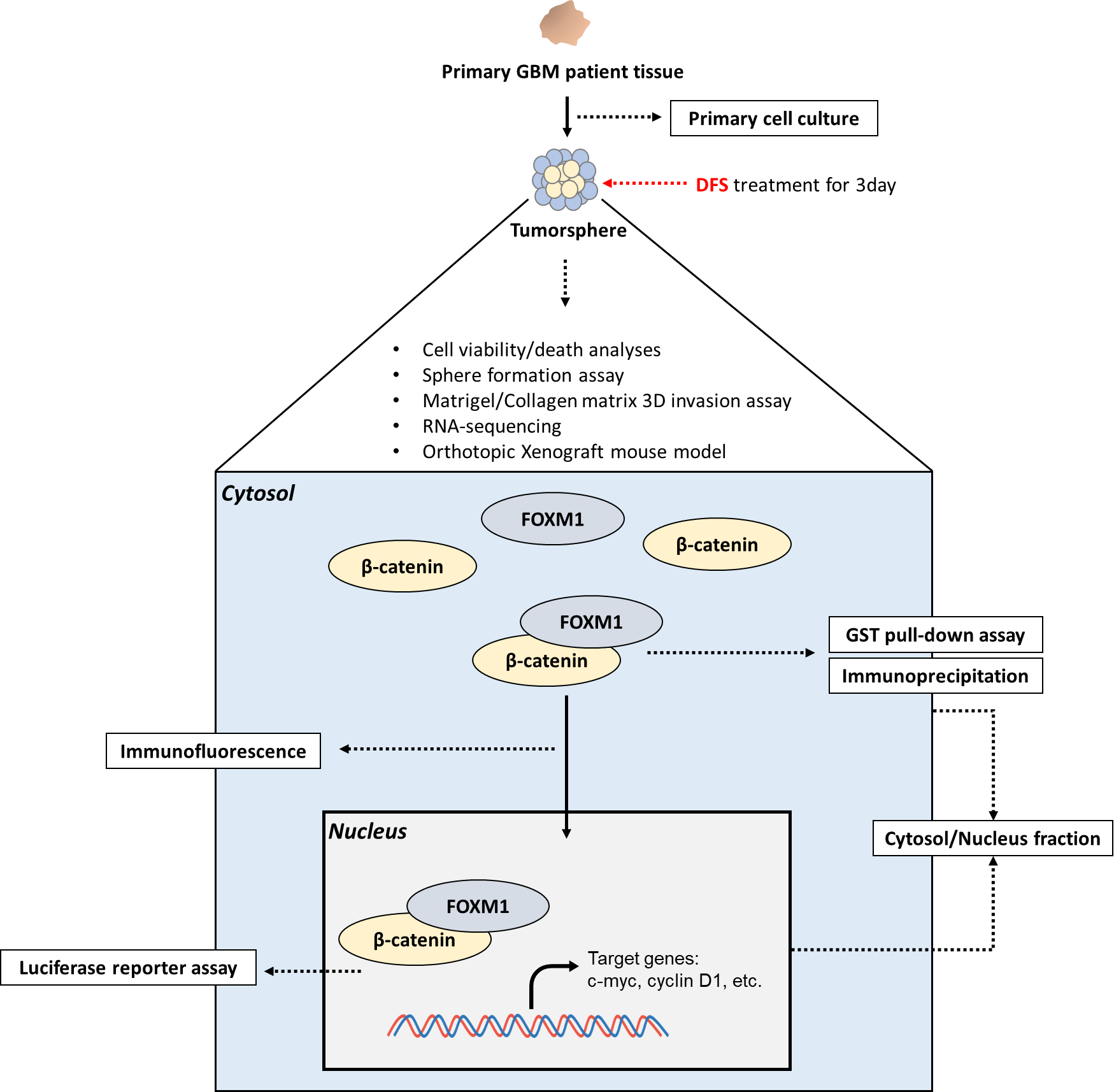


Figure S4. Graphical abstract. Tumorspheres(TSs) were obtained by performing cell culture from primary GBM patient tissue obtained through tumor resection surgery. Cell viability/death analyses, sphere formation assay, Matrigel/collagen matrix 3D invasion assay, RNA-sequencing, and orthotopic xenograft mouse model experiments were performed using obtained TSs. Experiments to investigate the inhibitory action of DFS at each step, immunoprecipitation to confirm FOXM1/β-catenin interaction, and immunofluorescence to confirm nuclear translocation of β-catenin were performed. Furthermore, cytosol/nuclear fraction to quantitatively compare β-catenin in cytosol and nucleus, and Luciferase reporter assay to confirmβ-catenin/TCF signaling activity were also performed.


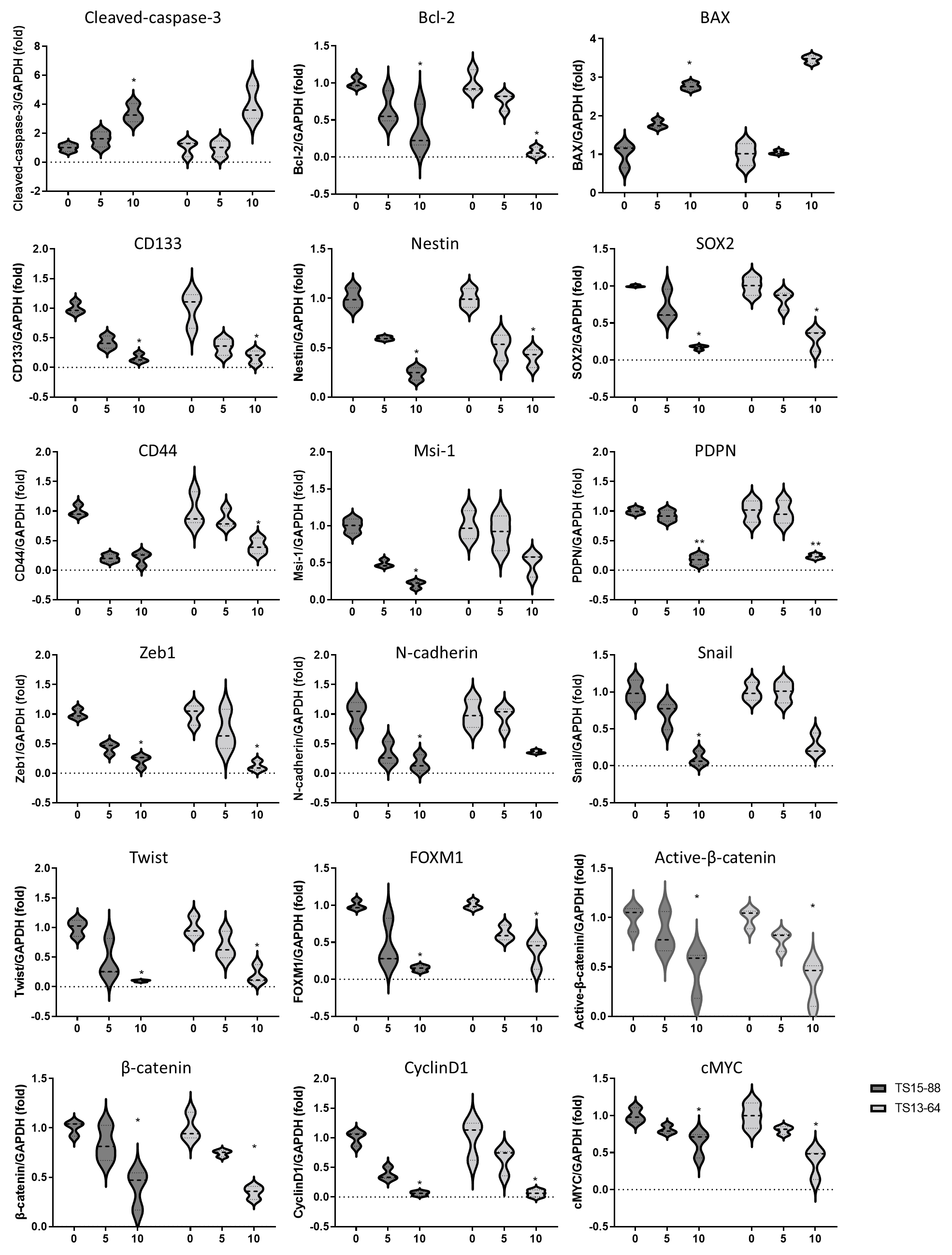


Figure S5. Violin plot representing the quantification of relative protein expression normalized with GAPDH.


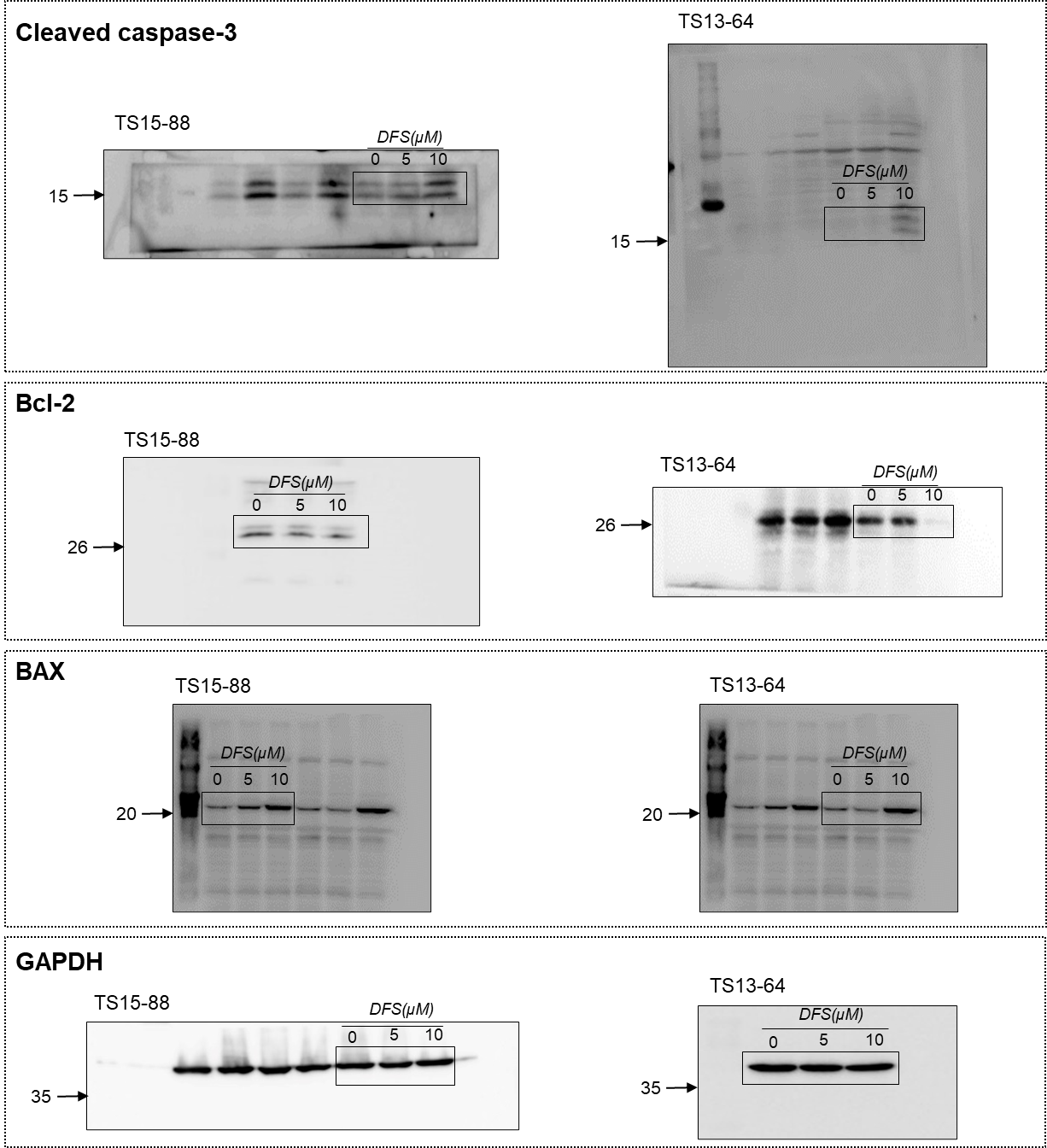


Figure S6a. original image of figure 1G.


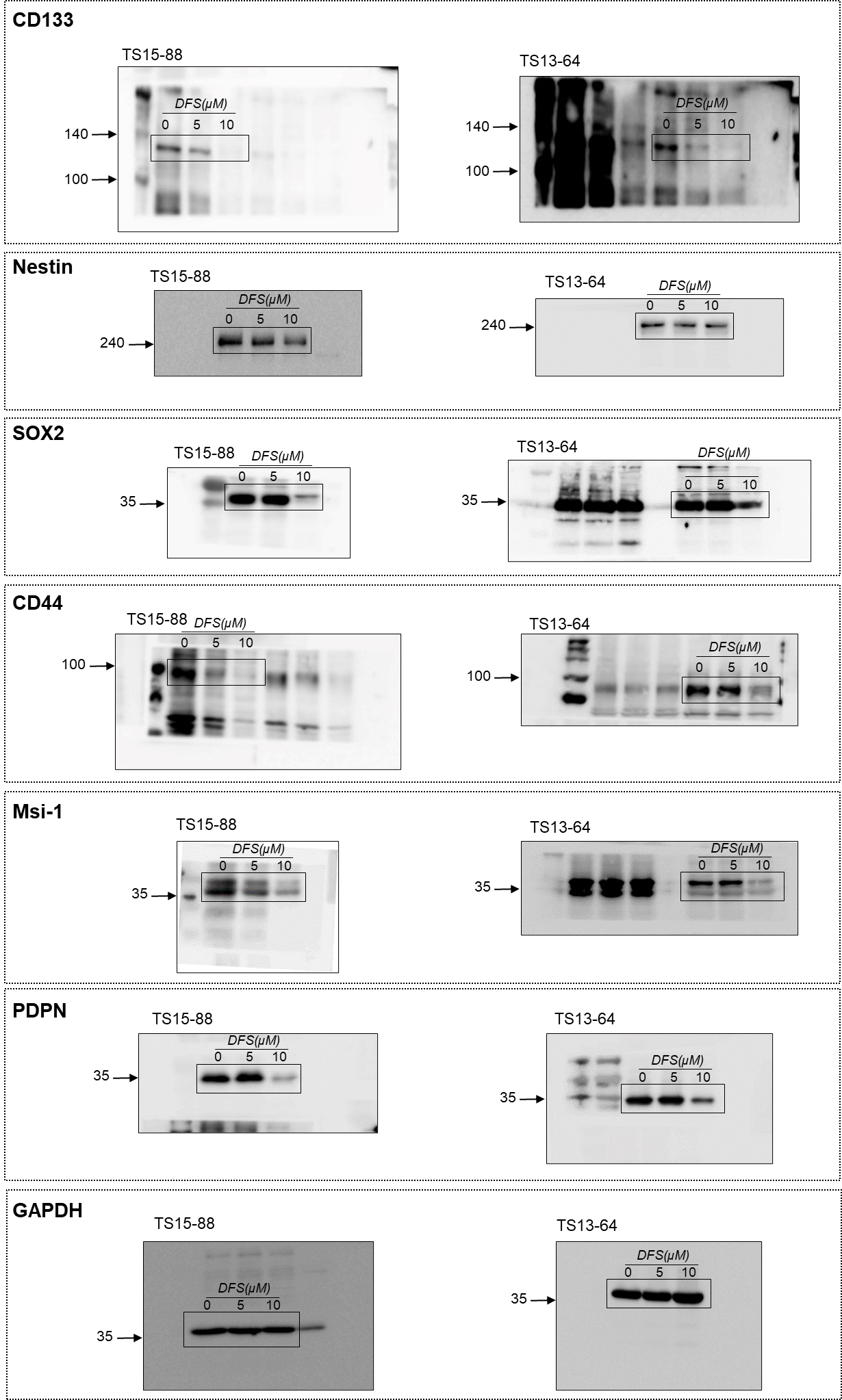


Figure S6b. original image of figure 2B.


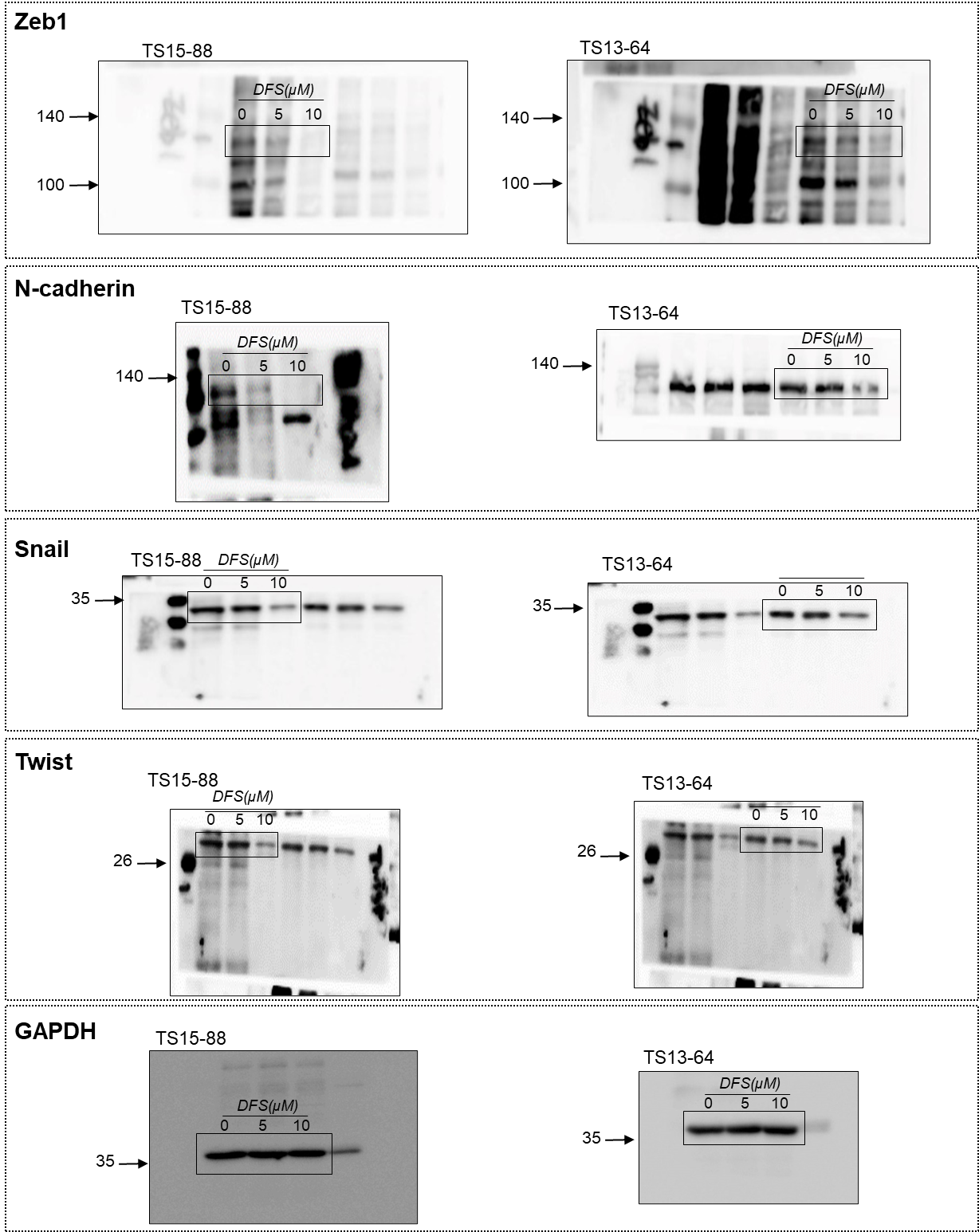


Figure S6c. original image of figure 2D.


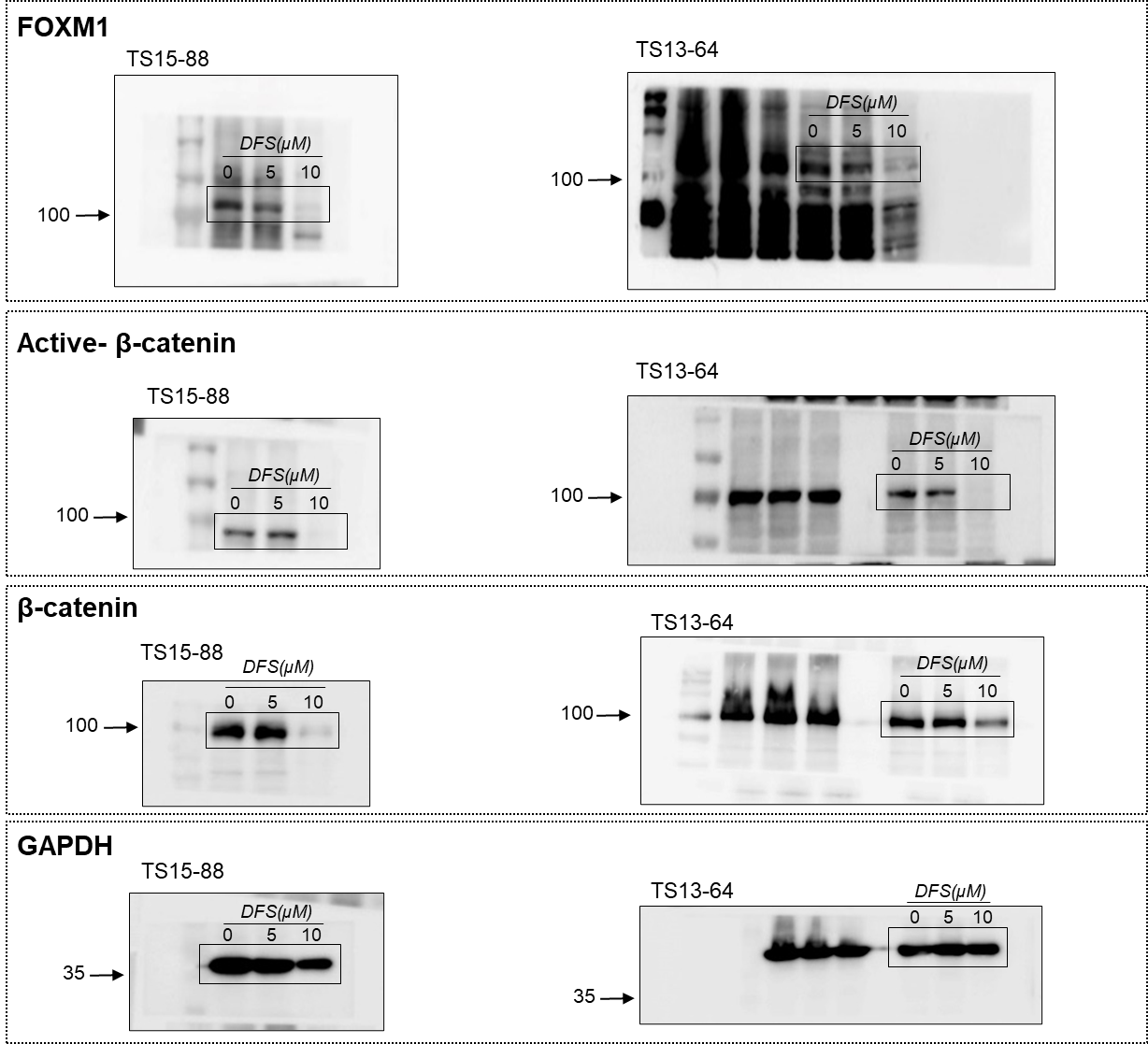


Figure S6d. original image of figure 3A.

**
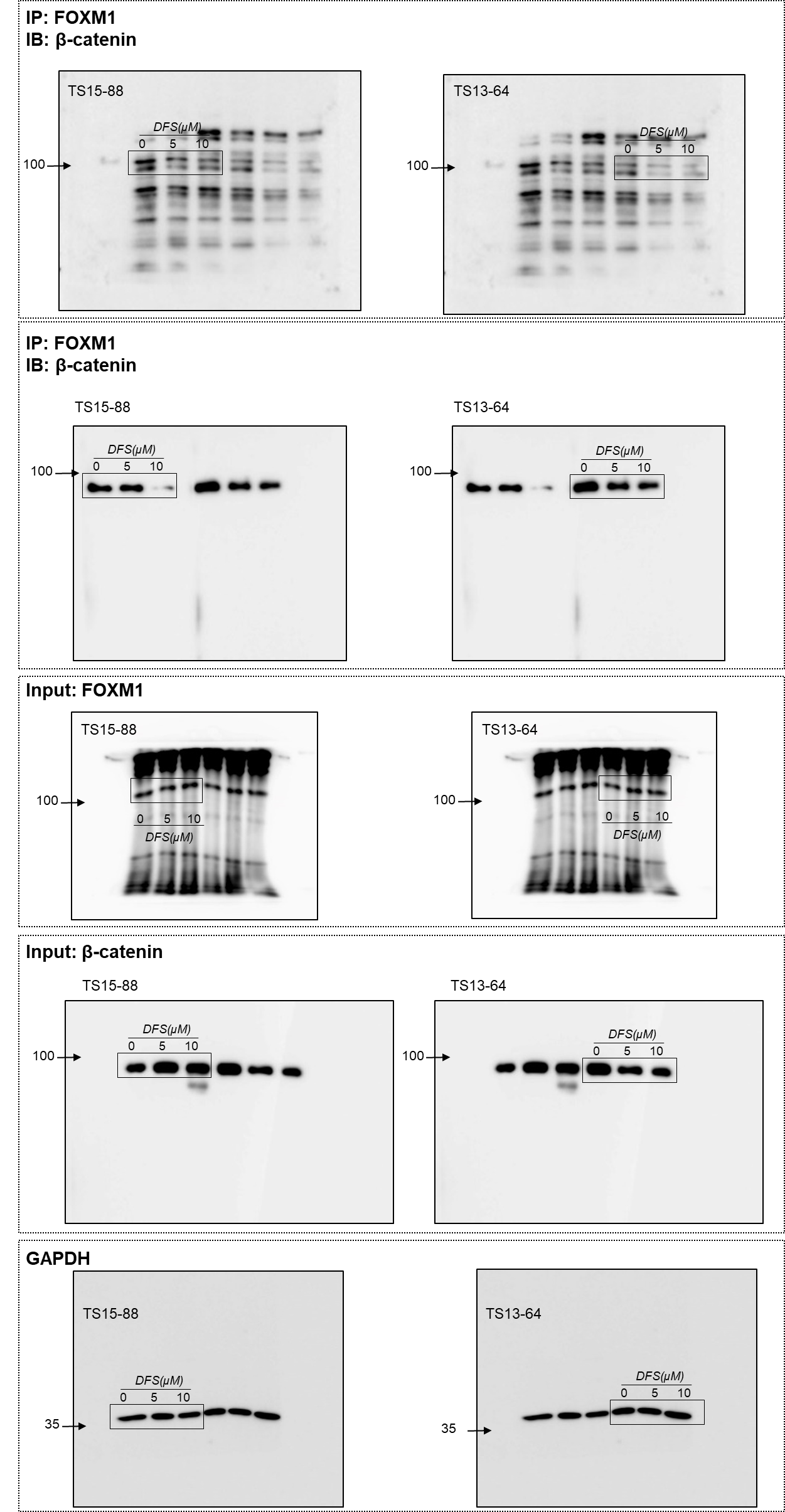
**

Figure S6e. original image of figure 3B.


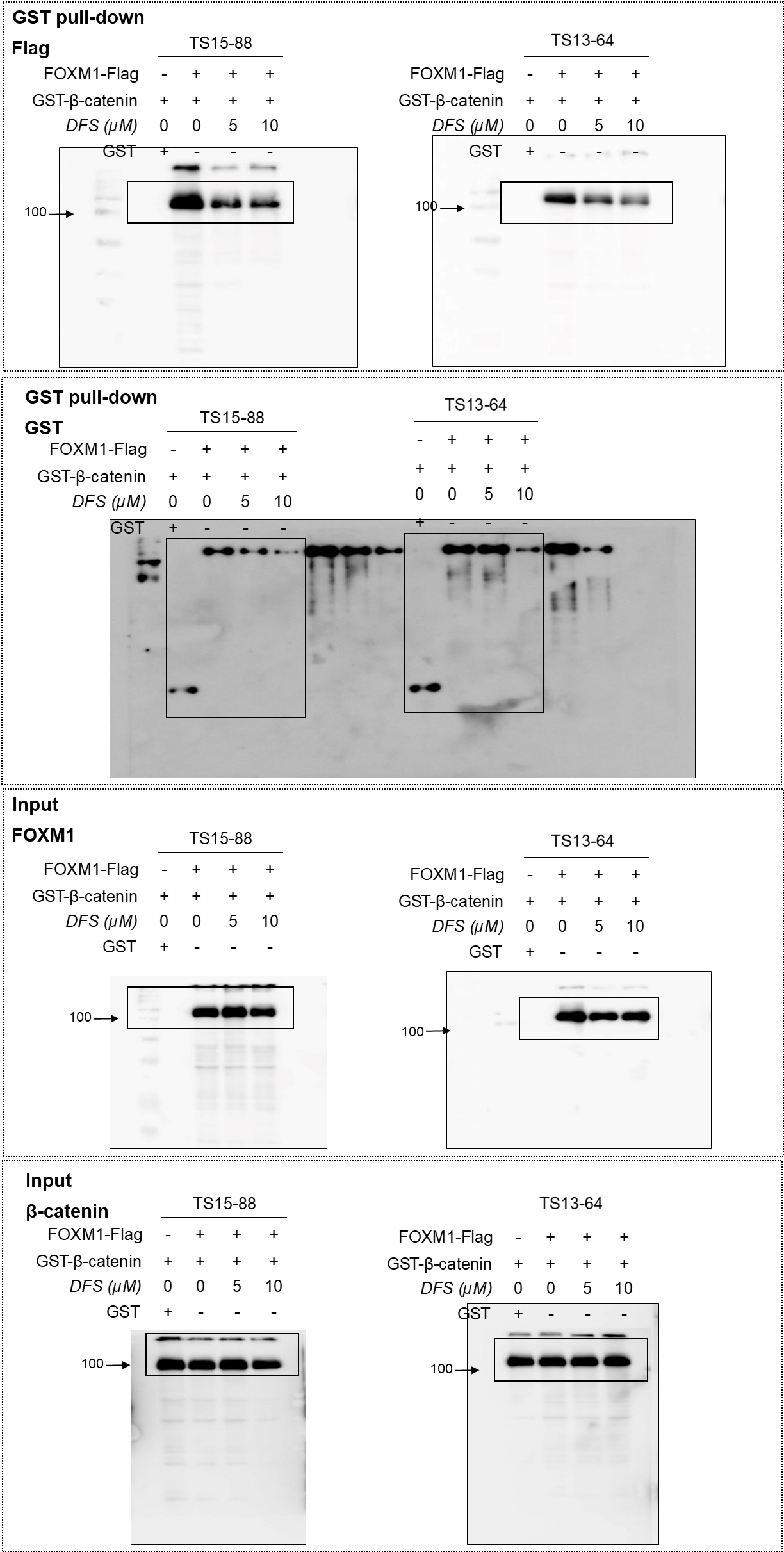


Figure S6e. original image of figure 3C.

**
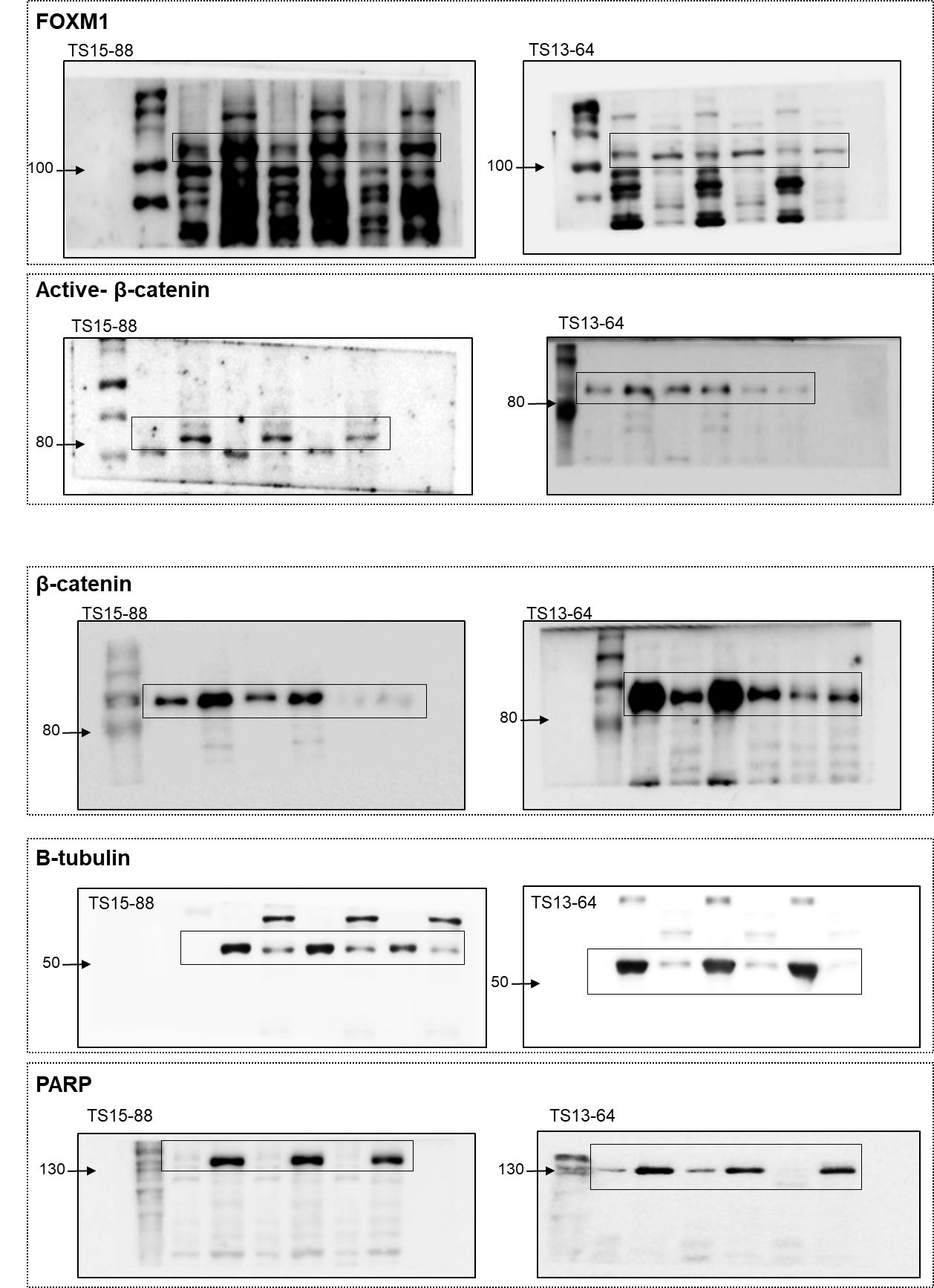
**

Figure S6f. original image of figure 3E.


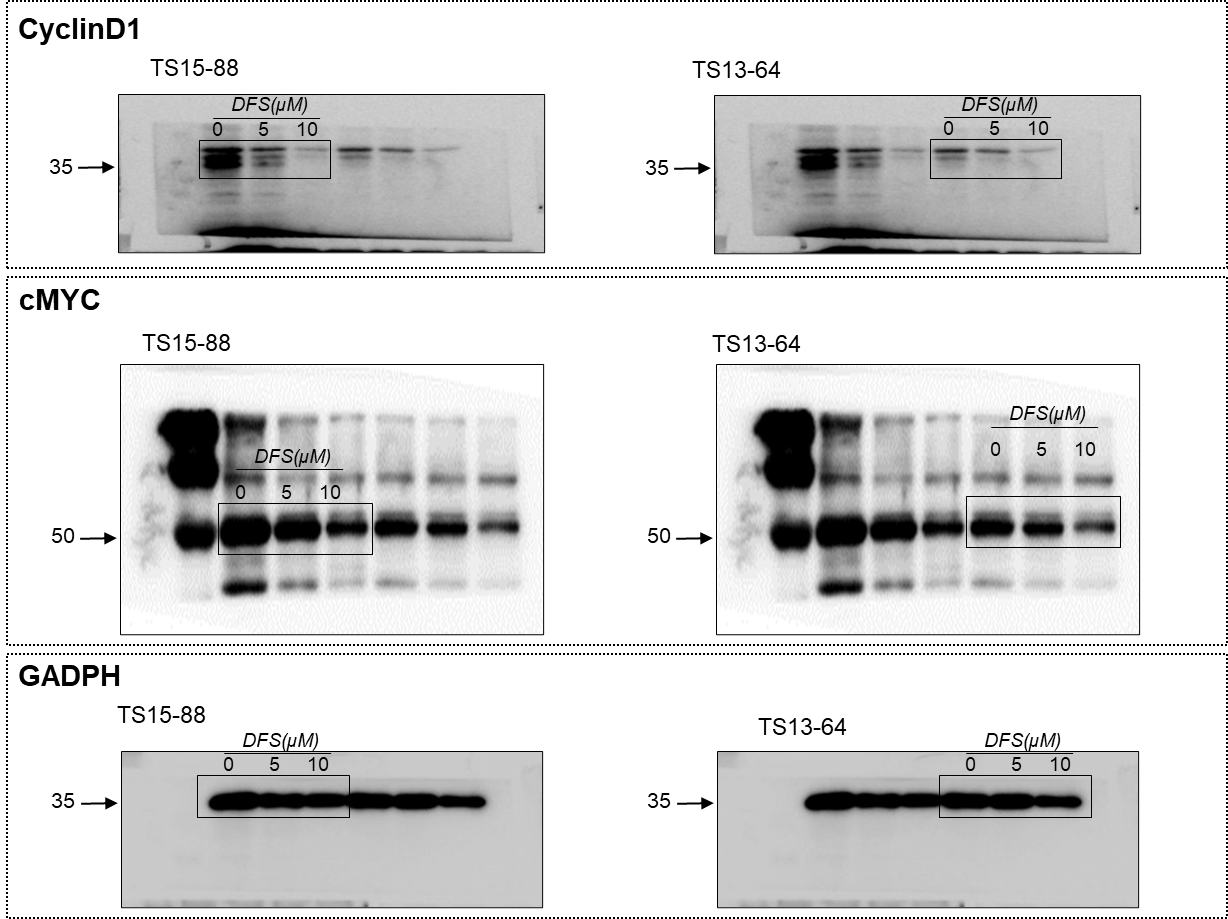


Figure S6g. original image of figure 3G.


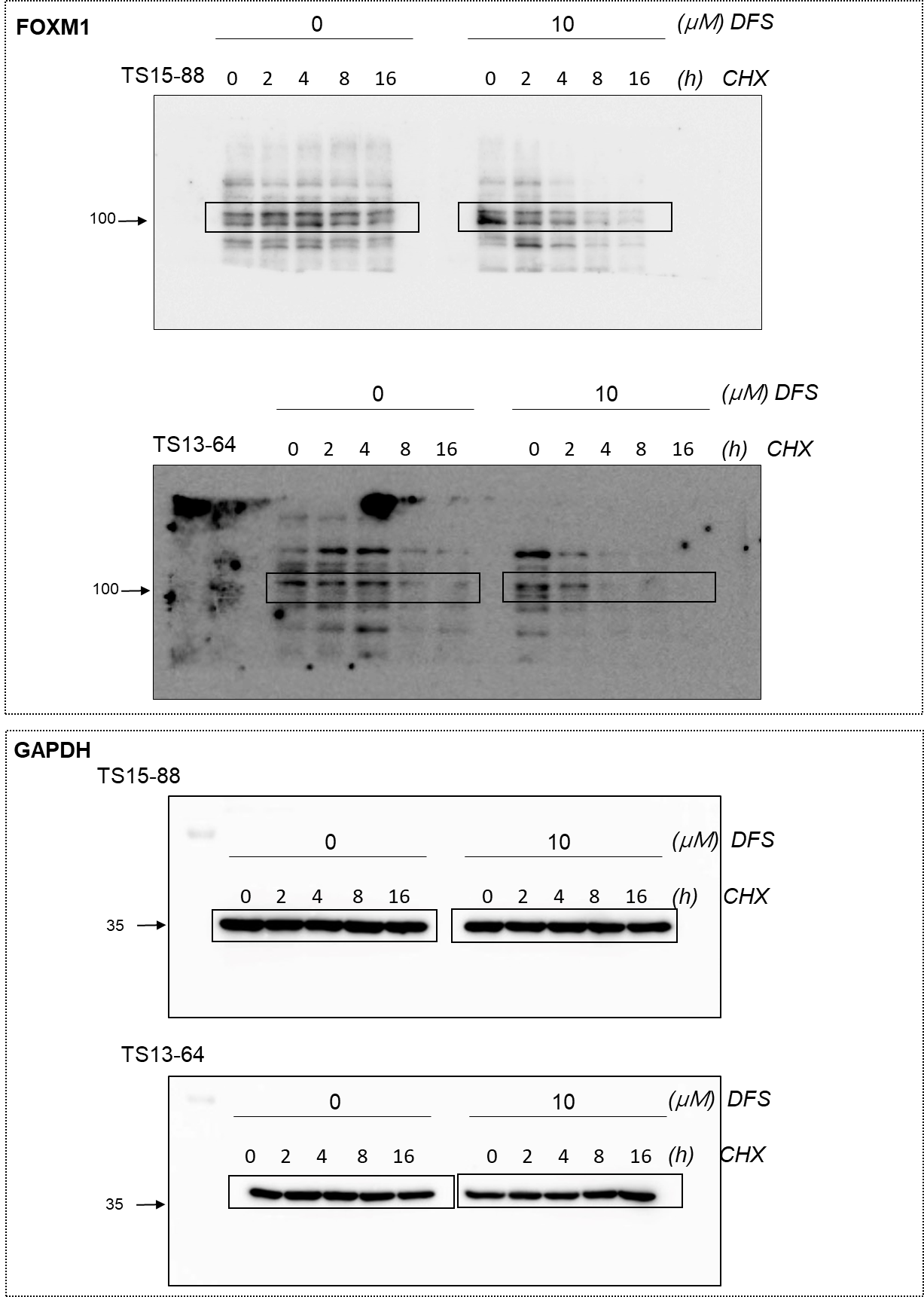


Figure S6h. original image of figure S3.
